# Environmental enrichment reduces restricted repetitive behavior by altering gray matter microstructure

**DOI:** 10.1371/journal.pone.0307290

**Published:** 2024-07-31

**Authors:** Anna L. Farmer, Marcelo Febo, Bradley J. Wilkes, Mark H. Lewis

**Affiliations:** 1 Department of Psychology, University of Florida, Gainesville, Florida, United States of America; 2 Department of Psychiatry, University of Florida, Gainesville, Florida, United States of America; 3 Department of Applied Physiology and Kinesiology, University of Florida, Gainesville, Florida, United States of America; Indian Institute of Technology Indore, INDIA

## Abstract

Restricted, repetitive behaviors are common symptoms in neurodevelopmental disorders including autism spectrum disorder. Despite being associated with poor developmental outcomes, repetitive behaviors remain poorly understood and have limited treatment options. Environmental enrichment attenuates the development of repetitive behaviors, but the exact mechanisms remain obscure. Using the C58 mouse model of repetitive behavior, we performed diffusion tensor imaging to examine microstructural alterations associated with the development of repetitive behavior and its attenuation by environmental enrichment. The C57BL/6 mouse strain, which displays little or no repetitive behavior, was used as a control group. We observed widespread differences in diffusion metrics between C58 mice and C57BL/6 mice. In juvenile C58 mice, repetitive motor behavior displayed strong negative correlations with fractional anisotropy in multiple gray matter regions, whereas in young adult C58 mice, high repetitive motor behavior was most strongly associated with lower fractional anisotropy and higher radial diffusivity in the striatum. Environmental enrichment increased fractional anisotropy and axial diffusivity throughout gray matter regions in the brains of juvenile C58 mice and overlapped predominantly with cerebellar and sensory regions associated with repetitive behavior. Our results suggest environmental enrichment reduces repetitive behavior development by altering gray matter microstructure in the cerebellum, medial entorhinal cortex, and sensory processing regions in juvenile C58 mice. Under standard laboratory conditions, early pathology in these regions appears to contribute to later striatal and white matter dysfunction in adult C58 mice. Future studies should examine the role these regions play in the development of repetitive behavior and the relationship between sensory processing and cerebellar deficits and repetitive behavior.

## Introduction

Restricted repetitive behaviors (RRB), commonly observed in autism spectrum disorder (ASD) and other neurodevelopmental disorders, encompass a wide range of inflexible behavior patterns including motor stereotypies, self-injurious behaviors, restricted interests, resistance to change, compulsive, and ritualistic behaviors [1, 2]. Despite evidence that these behaviors can be detrimental to social and cognitive development, RRB remain poorly understood and currently lack effective biomedical treatments [3, 4]. Interestingly, environmental enrichment (EE), a research paradigm in which animals are exposed to environments rich in physical, social, and cognitive stimulation, has been demonstrated to reduce repetitive behaviors in a variety of animal disease models [5–10]. Recent research also suggests that human treatments that provide sensory enrichment reduce autism symptoms including RRB [11–13].

Although numerous studies have documented brain changes after exposure to EE, the specific alterations responsible for EE’s attenuation of RRB are largely unknown [reviewed by 14]. Of the few studies that have examined the neural mechanisms behind EE’s reduction of stereotypies, most suggest that EE may prevent RRB development by increasing neurotrophin levels and synaptic plasticity within the indirect basal ganglia pathway and other basal ganglia regions [14–17]. For example, Lewis et al. [15] observed that C58 mice, an animal model of RRB, raised in EE displayed greater dendritic spine density and increased expression of genes associated with synaptic plasticity in the subthalamic nucleus than standard-housed (SH) control animals. Similar EE-related increases in dendritic spine density and arborization have also been found in deer mice, another model of RRB, in the striatum, subthalamic nucleus, and motor cortex [16, 18]. RRB reduction in enriched environments has also been connected to increases in BDNF in the striatum of deer mice, as well as in the prefrontal cortex and hippocampus of Fmr1 knockout mice, a rodent model of fragile X syndrome [17, 19]. However, the current understanding of how EE reduces RRB remains poorly understood and is limited by the relatively few brain regions and neurocellular mechanisms investigated to date [14].

Diffusion tensor imaging (DTI) is a type of diffusion weighted imaging (DWI) used to examine white matter microstructural integrity in neurodevelopmental and neuropsychiatric disorders [20, 21]. Increasingly, DTI metrics are being used as indicators of pathology in gray matter [22–24]. Within white matter, these metrics predominantly indicate axon structural integrity and myelination but can also reflect other neural processes such as inflammation or differences in axon density or diameter [20, 21, 25]. Within gray matter, diffusion metrics have been used as neural markers of neurodegeneration, dendritic complexity, and inflammation, but can also indicate changes in glial density and myelination of axons within gray matter [22, 26–29].

Several animal studies have used DTI to examine brain microstructural differences in rodent models of autism or RRB but few have included gray matter or identified correlations with RRB. To our knowledge, no published DTI studies have examined the microstructural differences underlying EE’s attenuation of RRB. Dodero et al. [30] and Ellegood et al. [31] used DTI to examine differences between BTBR mice, a rodent model exhibiting autism symptoms including RRB, and control mouse strains, but did not examine the relationship between DTI measures and RRB. In contrast, Wilkes et al. [32] used structural MRI and DTI to examine volumetric and microstructural differences between C58 and C57 mice and their relationship to RRB but did not examine EE effects. Several human studies have also used DTI to examine the relationship between diffusion metrics and autism symptom severity but have not examined these relationships in the context of an enriched environment [33–35]. In this study, we used DTI to identify brain regions and neural circuits important in RRB and underlying EE’s attenuation of RRB in an animal model of RRB. We also examined how EE alters neurocircuitry during early development. We hypothesized that both RRB and its attenuation by EE would result from microstructural alterations in basal ganglia and cerebellar circuits, particularly within the indirect basal ganglia pathway, which have previously been associated with RRB in MRI volumetric studies and earlier experimental studies of RRB in the C58 animal model [15, 36].

## Materials and methods

All animal experiments were conducted in accordance with National Institutes of Health policies and were approved by the Institutional Animal Care and Use Committee at the University of Florida (protocol # 202011229) to ensure the ethical and humane treatment of study animals.

### Animal housing

#### Adult study

To examine brain-behavior relationships in young adult mice, male and female C58 mice (n = 43, 19 females, 24 males) and C57BL/6 (C57) control mice (n = 39, 23 females, 16 males) were weaned on postnatal day 21 and assigned to standard housing (SH) or housing with environmental enrichment (EE) with the goal of balancing sex between housing conditions. C58 mice are an inbred mouse strain that naturally exhibit a high frequency of repetitive motor behavior, whereas C57 mice, a closely related inbred mouse strain, do not typically exhibit repetitive behaviors under standard or EE housing conditions [9, 37]. SH consisted of three to six same-sex mice per shoebox-style plastic cage (29 x 18 x 13 cm) with bedding and two nestlets available for nest construction. EE consisted of three to six same-sex mice housed in a large dog kennels (122 x 81 x 89 cm) with two additional levels connected by ramps, two running wheels, a shelter, bedding, 4 nestlets, habitrail tubes, and plastic toys that were rotated every two weeks to provide novelty. Both groups had continuous access to food and water and were kept in a room maintained on a 12:12 light dark cycle with a temperature range of 70–75 ˚F. To provide foraging opportunities, EE kennels had bird seed scattered throughout the kennels (2 oz/week), whereas the SH group had bird seed (2 oz/week) provided in the cage corner as a food supplement. Animals were housed in their respective cages for 6 weeks (42 days) until the start of behavioral testing. EE animals were transferred to an enriched standard cage at the beginning of behavioral testing, which consisted of a shoebox-style plastic cage (29 x 18 x 13 cm) with bedding and two nestlets containing a small running wheel with a plastic hut, a habitrail tube, and one toy.

#### Developmental study

To examine brain-behavior relationships during development, a younger cohort of juvenile C58 mice was examined in a second experiment. Male and female C58 mice (n = 22, 12 females, 10 males) were weaned on postnatal day 21 and assigned to SH or EE housing as described above. EE and SH housing was as described above for the adult study with the exception that animals were housed in their respective cages for 3 weeks (21 days) before behavioral testing and transfer to the enriched standard cage.

#### Behavioral assessment

Repetitive motor behaviors were assessed for adult study animals at 6 weeks post-weaning and developmental study animals at 3 weeks post-weaning. C58 mice demonstrate adult patterns of stereotypy by 2 weeks post-weaning but do not display an adult frequency of RRB until 5 weeks post-weaning [9]. The timing of behavioral testing was chosen to coincide with the completion of these two stages of stereotypy development in C58 mice. Mice were placed individually in test cages, and the amount of jumping and somersaulting was quantified using automated photobeam arrays that measure vertical activity. Automated recordings were verified by trained observers who watched and counted vertical jumps in a subsample of videos of the behavioral testing. All testing was conducted during the 12-hour dark cycle with food and water available *ad lib* during testing sessions. The total number of repetitive motor behaviors per dark cycle was calculated for each animal.

### MRI acquisition

Following behavioral assessment, mice were anesthetized using a combination of isoflurane (3% for induction, 1–2% for setup, and 0.5% during scans) and dexmedetomidine (Dexdomitor, Zoetis; 0.1 mg/kg injected intraperitoneally 40 minutes before scans followed by a continuous subcutaneous infusion at 0.05 mg/kg/hr during scans). Isoflurane was delivered in combination with medical grade air (0.4 L/min) via a nosecone. Animals were scanned in the prone position in a custom-made cradle with a bite bar and tubes for warm-water circulation to maintain the animal’s temperature at 36–37°C. Respiration was monitored continuously using a pressure pad under the animal with lubricating eye ointment applied before scans to prevent corneal desiccation. MRI images were acquired at the Advanced Magnetic Resonance Imaging and Spectroscopy (AMRIS) facility at the University of Florida on an 11.1 T scanner (Magnex Scientific Ltd., Oxford, UK) with an Advance III Bruker Paravision 6.01 console (Bruker BioSpin, Billerica, MA) using a 2 cm x 2.5 cm custom-made quadrature surface transmit/receive coil (470.7 MHz). Following scans, mice were injected with an intraperitoneal injection of 0.1 mg/kg atipamezole (Antisedan, Zoetis) if they experienced difficulty recovering from anesthesia.

For each mouse, a T2-weighted anatomical (T2) and diffusion-weighted scan were acquired during the same session. The T2-weighted scan was a Turbo Rapid Acquisition with Refocused Echoes (TurboRARE) sequence with the following parameters: effective echo time (TE) = 41 ms, repetition time (TR) = 4 seconds, RARE factor = 16, number of averages = 12, field of view (FOV) of 15 mm x 15 mm x 12.6 mm, resolution of 58.59 μm x 58.59 μm x 900 μm, and a data matrix of 256 x 256 and 14 interleaved slices covering the entire brain from the olfactory bulb caudally towards the spinal cord (acquisition time 9 minutes 36 seconds). DWI scans were acquired using a four-shot 3D diffusion weighted spin echo sequence, with TR  =  4 seconds, echo time TE  =  16 ms, pulse duration (δ)  =  3 ms, pulse spacing (Δ)  =  8 ms, and 3 averages. The FOV was 17.5 mm x 15 mm x 16.25 mm with a 70 x 60 x 65 matrix and a slice thickness of 0.25 mm resulting in a 0.25 mm^3^ resolution. DWI scans used a single shell diffusion weighting scheme with a total of 20 diffusion directions including four b  =  0 s/mm2 volumes and twenty b  =  1,000 s/mm2 volumes. The DWI scan time was 19 minutes 12 seconds. Diffusion scans with poor cerebellar coverage (10 animals) or low scan quality (12 animals) were excluded from analyses. Scans were not acquired for seven animals with behavioral data. Of the remaining 16 juvenile and 59 adult animals (Table 1), an average of 1.64 scans (SD = 1.89, max = 6) were removed due to the presence of respiratory or gradient-induced artifacts.

**Table 1:** Demographics of mice included in behavioral assessments (and DTI scans) for each age cohort.

Age Group	Housing Type	C58 Females	C58 Males	C57 Females	C57 Males
Adult	EE	8 (7)	12 (9)	12 (8)	9 (6)
	SH	11 (9)	12 (8)	11 (8)	7 (4)
Juvenile	EE	6 (5)	4 (4)	Not included	Not included
	SH	6 (4)	6 (3)	Not included	Not included

EE = environmentally enriched housing. SH = standard housing.

### Diffusion-weighted image preprocessing

Diffusion-weighted images were preprocessed with MRtrix3 [38] and included the following preprocessing steps: noise estimation and denoising, removal of Gibbs ringing artifacts, correction for motion and eddy current distortions, and bias field correction. Susceptibility-induced EPI distortions were not corrected. Brain masks were created in MATLAB using Three-Dimensional Pulsed Coupled Neural Networks (PCNN3D) [39] and manually corrected in ITK-SNAP [40]. Following preprocessing, a diffusion tensor model was fit to each voxel followed by the calculation of fractional anisotropy (FA), mean diffusivity (MD), radial diffusivity (RD), and axial diffusivity (AD) maps for each subject. Following preprocessing, the B0 images for each subject were averaged together and the mean B0 image was bias field corrected using the N4BiasFieldCorrection command in ANTs [41]. The corrected mean B0 image was then registered to a mouse reference atlas (P56 Allen Brain Atlas) [42] using linear registration in FSL FLIRT [43] followed by non-linear registration in ANTs [44]. The transformation matrices from these registration steps were then applied to each subject’s diffusion metric maps to register them to the Allen Brain Atlas.

### Statistical analysis

#### Adult study

The total number of repetitive motor behaviors were compared between experimental groups in SPSS version 28.0.1.1 using a Kruskal-Wallis test followed by pairwise Bonferroni-corrected post hoc pairwise comparisons. A voxel-based approach was taken to examine diffusion metric differences across the entire brain. For the adult cohort, the effects of housing, inbred mouse strain, and sex on DTI metrics were evaluated using a voxelwise three-way between-subjects analysis of variance (ANOVA) in FSL randomise [45] using 500 random permutations. Statistically significant differences were then examined using post hoc t-tests. To determine brain regions with diffusion differences associated with repetitive motor behavior, we conducted a voxelwise correlation between repetitive motor scores and each DTI metric using FSL randomise and 500 permutations. To control for multiple comparisons, all voxelwise analyses used threshold-free cluster enhancement (TFCE) [46] and family-wise error rate (FWE) [47] correction of p-values to maintain an alpha level of 0.05. FSL Cluster was used on resulting significant voxels to determine cluster size and voxel coordinates for peak and local maximum signals within each cluster with corresponding brain regions identified by coordinate comparison with the P56 Allen Brain Atlas [48].

#### Developmental study

The total number of repetitive motor behaviors were compared between experimental groups as described in the adult study. The effects of housing and sex on DTI metrics were determined using a voxelwise two-way between subjects ANOVA in FSL randomise with 500 permutations. Statistically significant differences were examined by post hoc t-tests. To determine brain regions associated with repetitive behavior, we conducted a voxelwise correlation between repetitive motor scores and each DTI metric using FSL randomise and 500 permutations. To control for multiple comparisons, all voxelwise analyses were TFCE and FWE corrected. FSL Cluster was used to identify signal peaks in significant clusters as described in the adult study.

## Results

### Adult study: Six weeks post-weaning cohort

#### EE reduces repetitive motor behavior in adult female C58 mice

**Fig 1:**
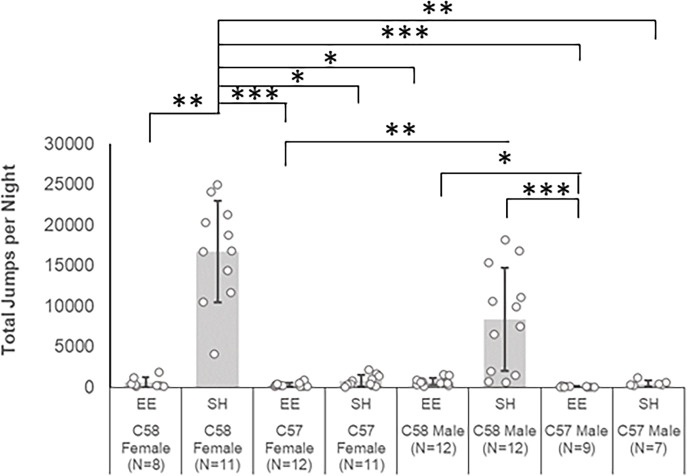
Mean number of repetitive motor behaviors exhibited by experimental groups in the adult cohort. *p < .05, **p < .01, ***p < .001. All p values are Bonferroni corrected. Error bars represent standard deviation.

A Kruskal-Wallis test determined that there were significant differences in repetitive motor behavior between experimental groups [χ^2^(7) = 57.63, p < 0.001; Fig 1]. Based on Bonferroni-corrected post hoc pairwise comparisons, SH female C58 mice exhibited a significantly (corrected p < 0.05) higher frequency of jumping behavior than all other groups except for SH male C58 mice. SH male C58 mice also had significantly greater repetitive behavior than EE C57 male (Z = 56.53, p < 0.001) and EE C57 female mice (Z = 36.83, p = 0.004), but interestingly was not significantly different from EE C58 male mice despite a much higher mean repetitive behavior ($\bar{x}\;$ = 8,366 vs. 666 jumps per night, respectively) due to the presence of four individuals with low RRB measures in this group. The EE C57 male mice also had significantly lower repetitive motor behavior than EE C58 male mice (Z = 33.53, p = 0.04).

#### Widespread microstructural differences in adult C58 mice

**Fig 2:**
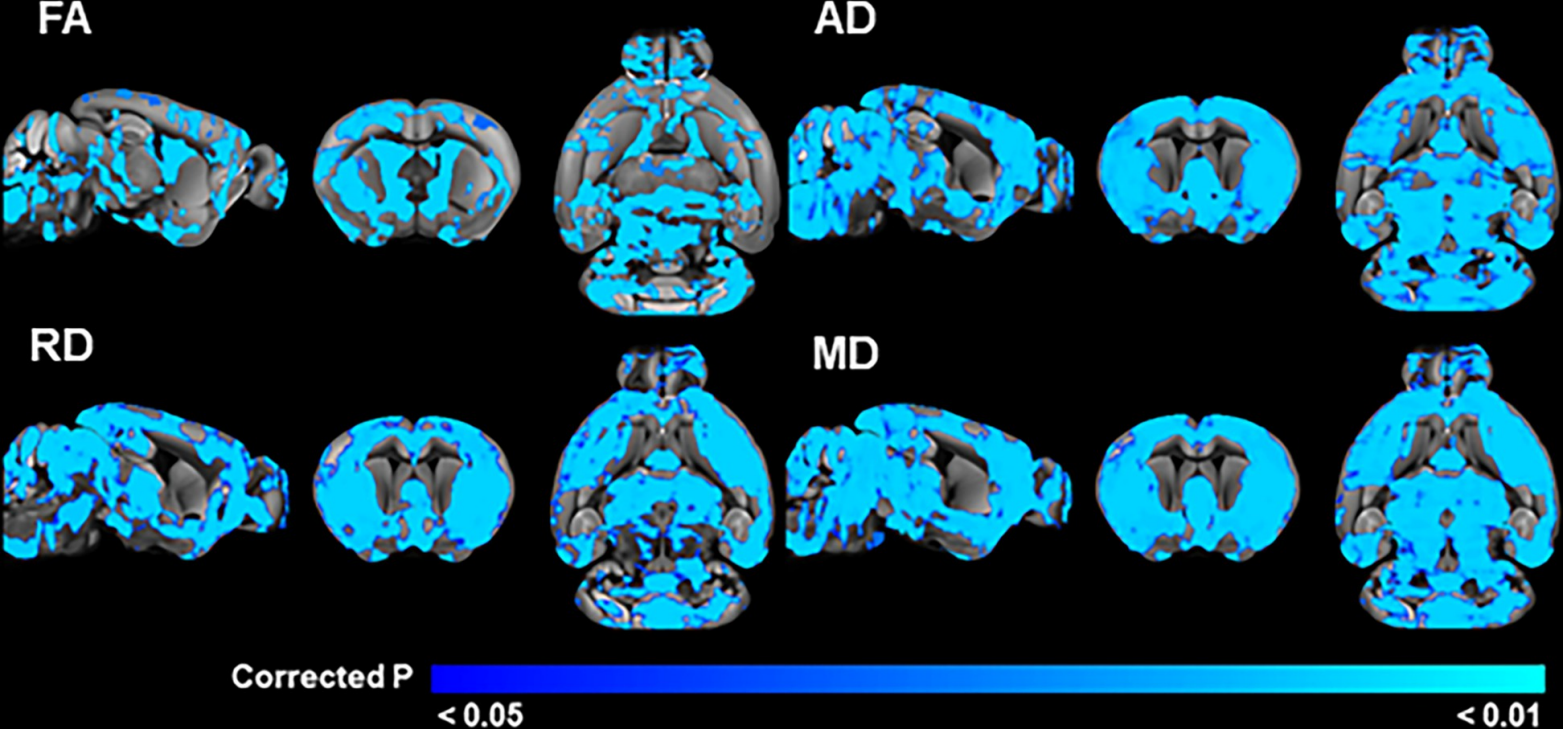
Brain regions with significant mouse strain differences in DTI measures in the adult cohort. Blue areas indicate regions where C58 mice have significantly (TFCE and FWE corrected p < 0.05) reduced values compared to C57 mice.

In the older, 6-week postweaning animals, the three-way ANOVA revealed a significant main effect of mouse strain for all DTI metrics. Post hoc t-tests revealed that all diffusion metrics were reduced in C58 mice compared to C57 mice in regions throughout the brain (Fig 2). For FA, these significant regions included clusters with signal peaks in multiple basal ganglia regions (striatum, substantia nigra pars reticulata, globus pallidus external segment), the optic tract, primary somatosensory cortex and other sensory processing regions, multiple thalamic regions involved in executive and motor functions, cortical and subcortical motor regions, and cerebellar motor regions and white matter tracts, as well as regions important in learning and memory, fear, and limbic processing (S1 Table in S1 Appendix). For AD, significant regions included clusters with signal peaks in the hypothalamic nuclei with roles in feeding, cortical sensory and motor regions, regions involved in social behaviors (medial amygdala), as well as cerebellar motor and cognitive regions (S2 Table in S1 Appendix). Regions with significantly reduced RD in C58 mice included clusters with signal peaks in hypothalamic regions important for feeding, homeostasis, hormonal regulation, and metabolism; cortical and subcortical sensory and motor regions; hippocampal regions; the medial entorhinal cortex; insular and retrosplenial cortices; and cerebellar regions involved in movement, coordination of eye movements, and higher cognitive functions (S3 Table in S1 Appendix). C58 mice had lower MD than C57 mice in clusters in gray and white matter regions with signal peaks in hypothalamic regions associated with feeding and metabolism, growth, and homeostasis; cerebellar regions involved in eye movement and higher cognitive functions; sensory and motor regions; and limbic regions (S4 Table in S1 Appendix).

#### Adult EE females have lower FA than SH females

**Fig 3:**
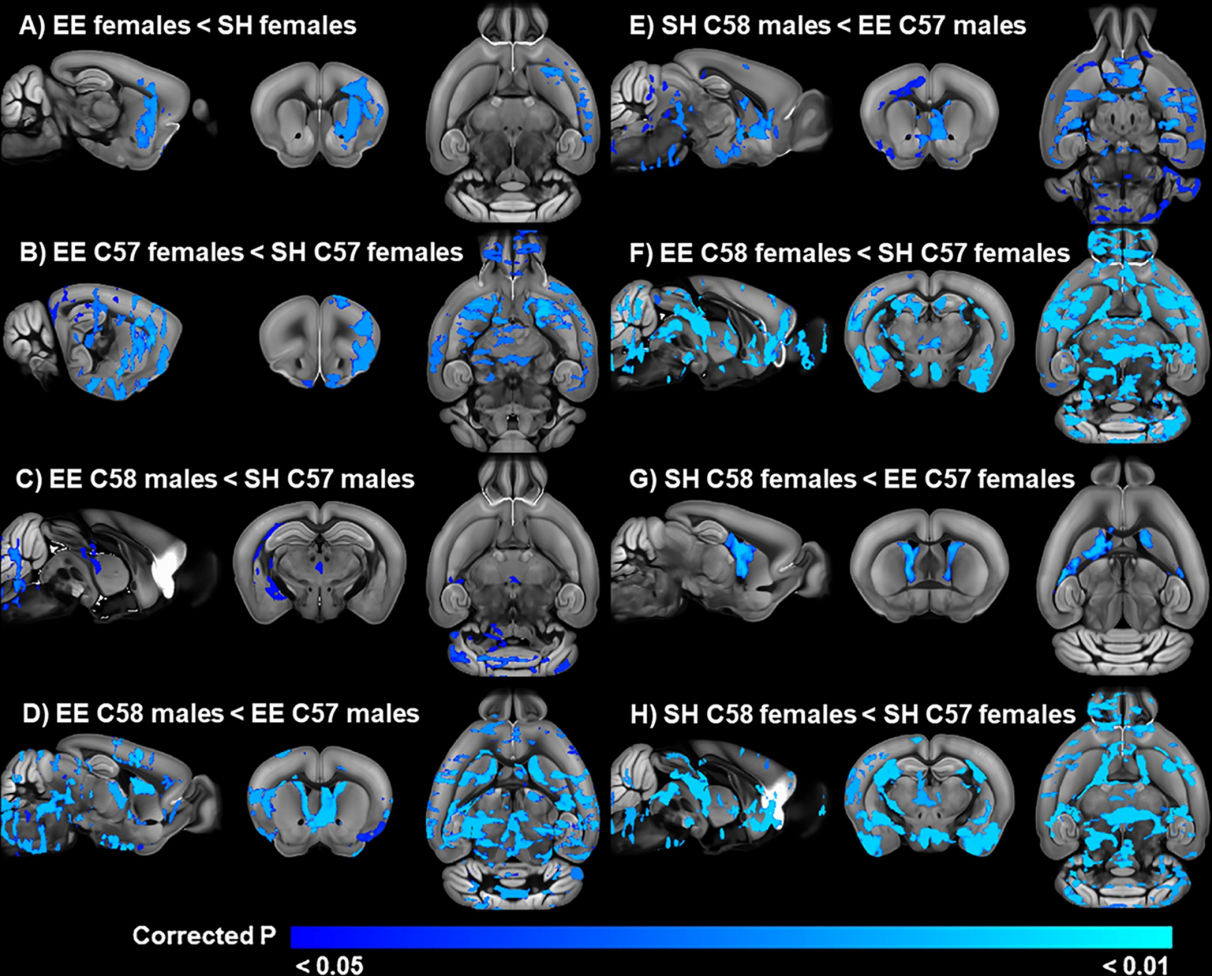
Relationship between EE and FA in the adult cohort. Significant A) sex by strain and B-H) sex by strain by housing interactions for FA in the adult cohort. Blue areas indicate regions with significantly (TFCE and FWE corrected p < 0.05) reduced FA.

The three-way ANOVA revealed a significant housing by sex interaction for FA and significant interaction effect of housing, strain, and sex for FA. Interestingly, housing main effects were not significant for FA or any other DTI metric. Post hoc t-tests to decompose the housing by sex effect for FA did not identify any significant differences between EE and SH males. In contrast, EE females had lower FA than SH females in a single cluster with signal peaks in the left agranular insular, primary motor, and primary somatosensory cortices (Fig 3A; S5 Table in S1 Appendix).

#### Significant housing by strain by sex interactions in FA in adult mice

Post hoc t-tests were conducted to determine the group differences responsible for the significant interaction of housing, strain, and sex on FA in the 3-way ANOVA. Interestingly, the group comparisons did not find any significant differences between SH and EE C58 female mice, despite observations of significantly decreased RRB in EE C58 female mice. Similarly, no significant differences were found between housing conditions in C58 males, nor between housing conditions in C57 male mice. In contrast, post hoc tests identified reduced FA in EE C57 females compared to SH C57 females localized to a single cluster with signal peaks in the right bed nucleus of the stria terminalis, right dorsolateral striatum, left agranular insula, right olfactory regions, and left thalamus (Fig 3B; S6 Table in S1 Appendix).

Post hoc t-tests also revealed that EE C58 males had lower FA than SH C57 males (Fig 3C). These significant regions included 3 clusters with signal peaks in the right cerebellum and brainstem, right medial amygdala nucleus, hippocampus, corpus callosum, optic radiation, and the third ventricle as the peak for the third cluster, suggesting some partial volume effects (S7 Table in S1 Appendix). EE C58 males also had lower FA than EE C57 males throughout the brain (Fig 3D). Significant regions included a single cluster with signal peaks in the left dorsomedial striatum and cerebellum, although the lateral ventricle was also included in this cluster suggesting some partial volume effects (S8 Table in S1 Appendix). In contrast, C58 SH males had lower FA than C57 EE males in a single cluster with signal peaks in the left central amygdala nucleus, left nucleus accumbens, right bed nucleus of the stria terminalis, and right pontine gray (Fig 3E; S9 Table in S1 Appendix).

Similar to the males, a post hoc t-test found that EE C58 females had lower FA than SH C57 females (Fig 3F) in a large number of predominantly gray matter subcortical and cortical regions (S10 Table in S1 Appendix). In contrast, SH C58 female mice had lower FA than EE C57 females in a single cluster with signal peaks in the left and right dorsomedial striatum, as well as a peak in the left lateral ventricle suggesting some influence of partial volume effects (Fig 3G; S11 Table in S1 Appendix). Similarly, SH C58 females had lower FA than SH C57 female mice in a number of gray and white matter regions with signal peaks in brain regions in the cerebellum, brainstem, basal ganglia, thalamus, amygdala, and cortex (Fig 3H; S12 Table in S1 Appendix). There were no other significant differences in FA between any other groups. There were also no main effects of housing, strain, sex, or interaction effects on MD, AD, or RD.

#### RRB in adult mice associated with lower FA and higher RD in striatum and white matter

**Fig 4:**
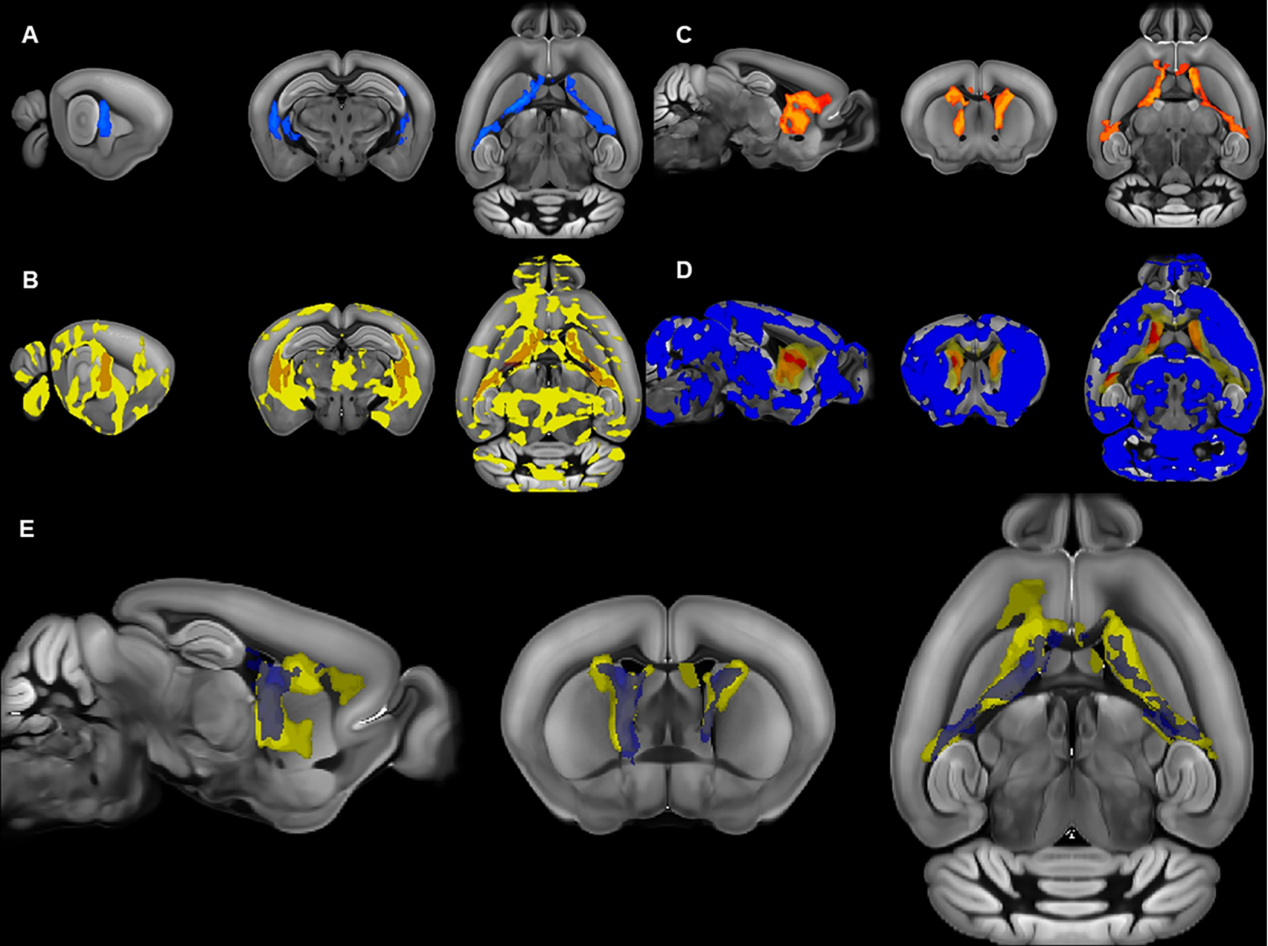
Relationship between diffusion metrics and repetitive motor behavior (RRB) in the adult cohort. A) Brain regions with a negative correlation (in blue) between FA and RRB. B) Overlap of areas with negative correlations between RRB and FA (in orange) and strain-associated decreases in FA in C58 mice (in yellow). C) Brain regions with a positive correlation (in red) between RD and RRB. D) Overlap (in orange) of brain regions with positive correlations between RRB and RD (in yellow) and almost significant (TFCE and FWE corrected p< 0.10) increases in RD in C58 mice compared to C57 mice (in red). Brain regions with RD correlations with RRB do not overlap with regions with significantly reduced RD in C58 mice (in blue). E) Overlap of brain regions with correlations between RD and RRB (yellow) and regions with correlations between FA and RRB (blue). All regions displayed are statistically significant (TFCE and FWE corrected p<0.05) unless otherwise indicated.

**Fig 5:**
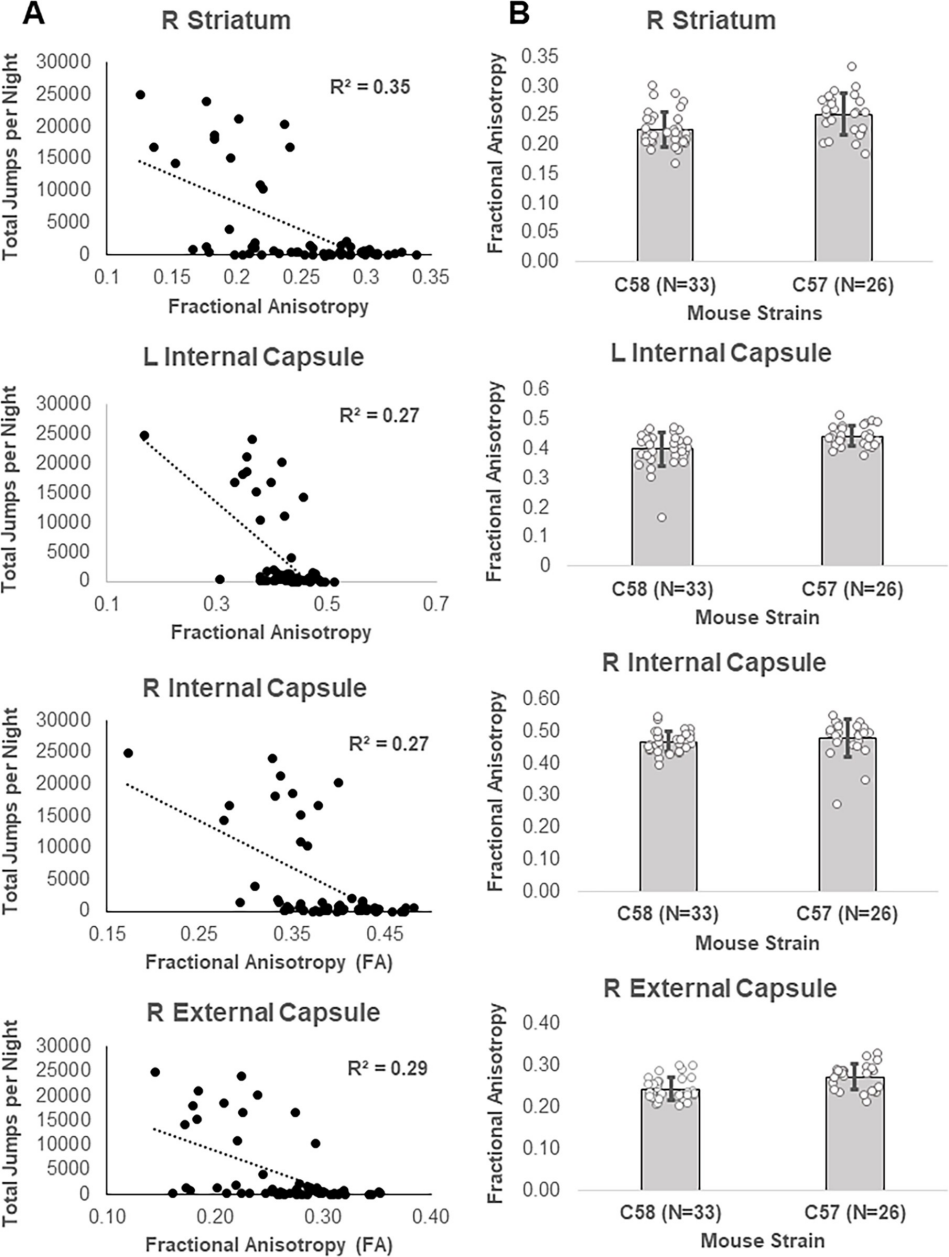
Regions with significant correlations between RRB and FA and FA strain differences in adult mice. A) Correlation between RRB and FA by brain region. B) FA differences between mouse strains by brain region.

In the adult cohort, there was a significant (TFCE and FWE corrected p < 0.05) negative correlation between repetitive motor behavior scores and FA (higher repetitive motor behavior was associated with lower FA) in several gray and white matter regions (Figs 4A and 5) included in a single cluster with signal peaks in the left and right internal capsule, as well as the right external capsule and caudal striatum (S13 Table in S1 Appendix). Brain regions with negative correlations between FA and RRB showed almost complete overlap with areas with strain differences between C58 and C57 mice (Fig 4B).

**Fig 6:**
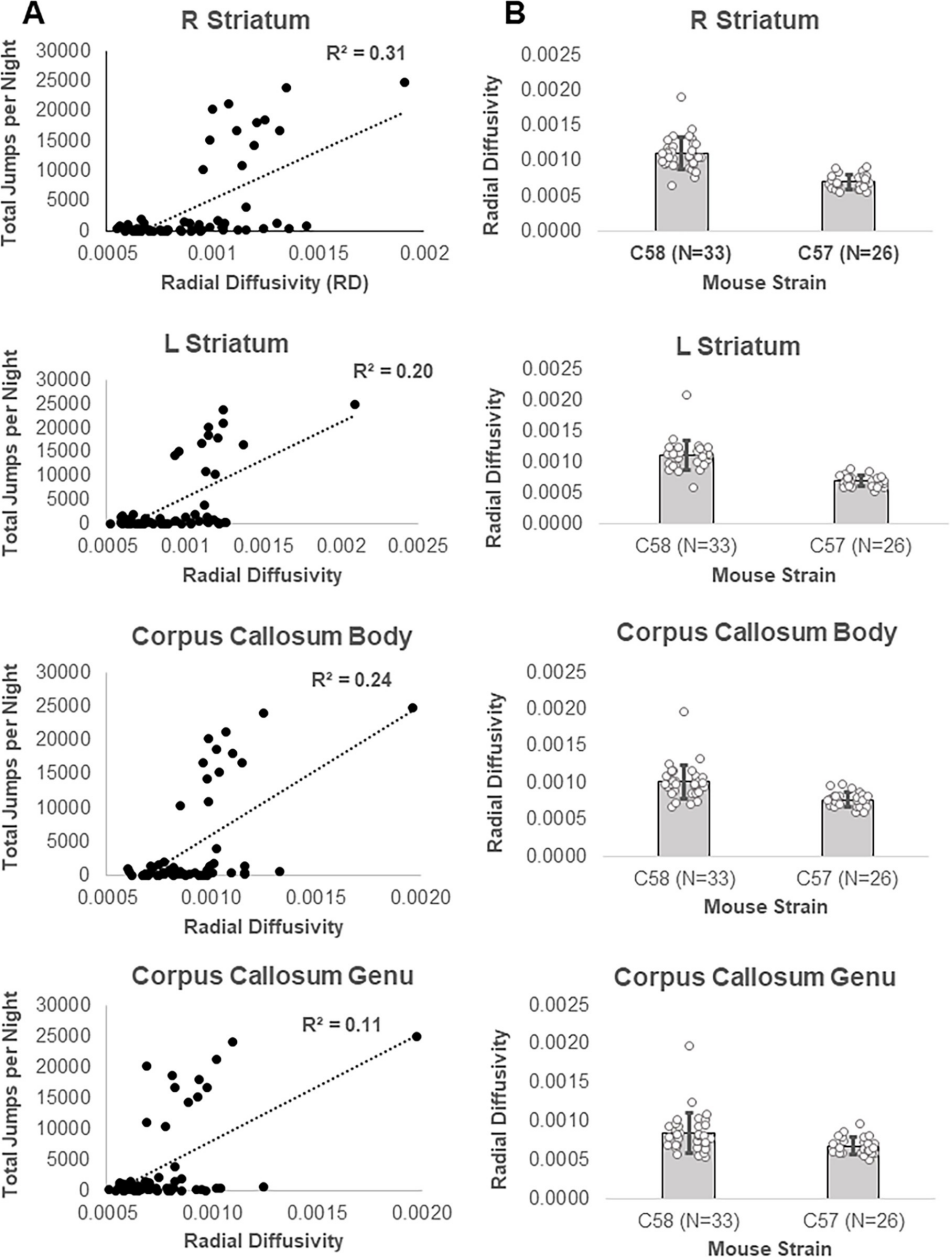
Relationship between correlations between RRB and RD and RD strain differences in adult mice. A) Correlation between RRB and RD by brain region. B) RD differences between mouse strains by brain region.

There was also a significant (TFCE and FWE corrected p < 0.05) positive correlation between repetitive motor behavior scores and RD (higher repetitive motor behavior was associated with higher RD) in a single cluster with signal peaks in the left and right striatum and body and genu of the corpus callosum (Fig 4C, S14 Table in S1 Appendix). These brain regions showed substantial overlap with the brain regions that had nearly significant increases in RD in the C58 mouse strain (left and right striatum, corpus callosum) but did not overlap with brain regions that showed significant decreases in RD in the C58 mouse strain (Figs 4D and 6). Brain regions with a positive correlation between RD and RRB and also showed considerable overlap with brain regions displaying a negative correlation between RRB and FA (Fig 4E).

### Developmental study: Three weeks post-weaning cohort

#### Repetitive motor behavior in juvenile mice

**Fig 7:**
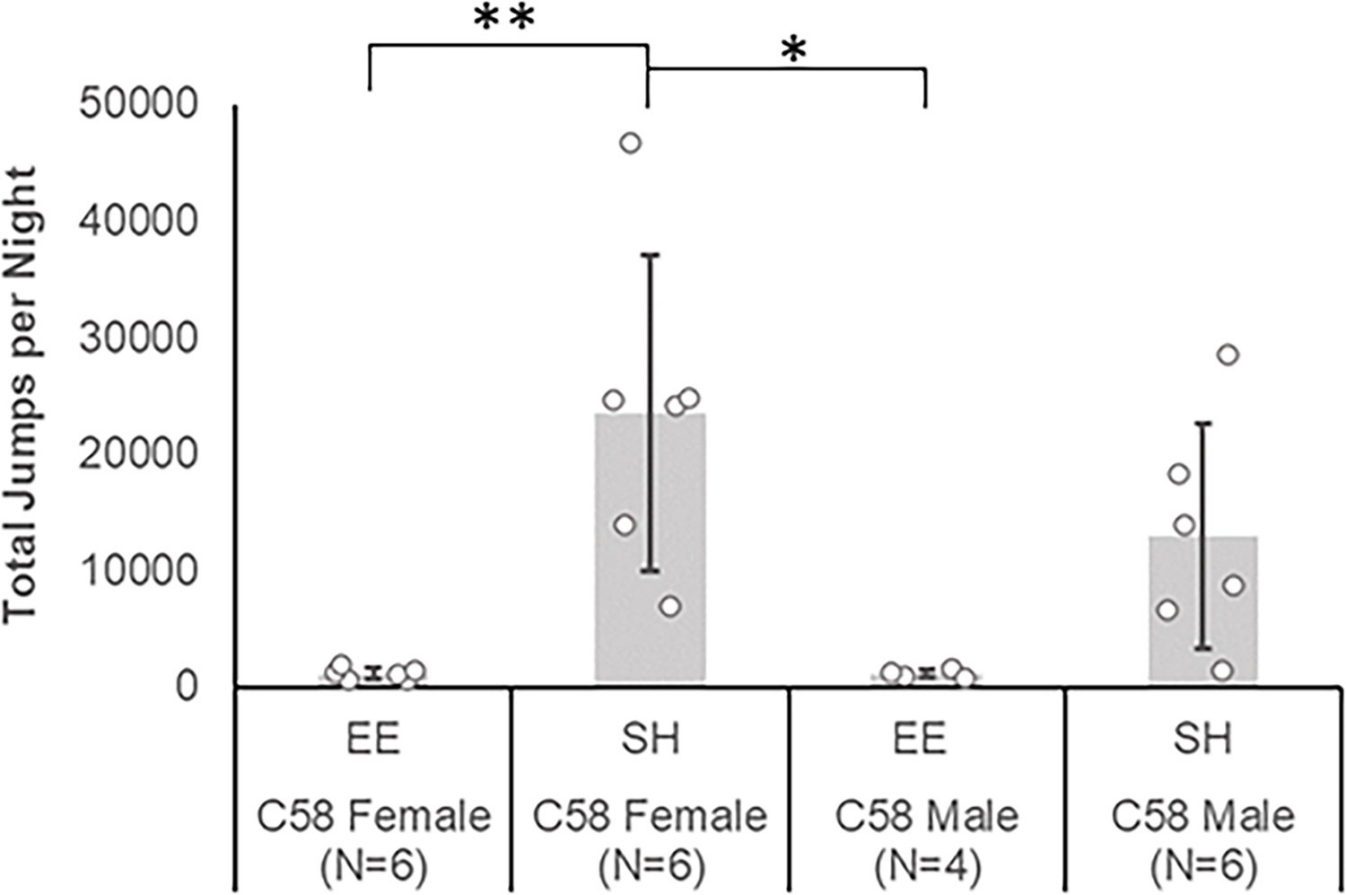
Mean number of repetitive motor behaviors exhibited by experimental groups in the juvenile cohort. *p < .05, **p < .01. All p values are Bonferroni corrected. Error bars represent standard deviation.

A Kruskal-Wallis test determined that there were significant differences in repetitive motor behavior between experimental groups [χ^2^(3) = 15.00, p = 0.002; Fig 7] in the juvenile cohort. Based on Bonferroni-corrected post hoc pairwise comparisons, the SH C58 female mice had significantly greater (corrected p < 0.05) repetitive motor behavior than EE C58 female mice (Z = -12.33, p = 0.006) and EE C58 male mice (Z = 12.00, p = 0.03). The repetitive motor behavior of SH C58 male mice was not significantly different from other experimental groups despite having a relatively high mean ($\bar{x}$ = 12,919) due to an individual with a low RRB measure in this group.

#### EE induces widespread increases in gray matter FA and AD in juvenile C58 mice

**Fig 8:**
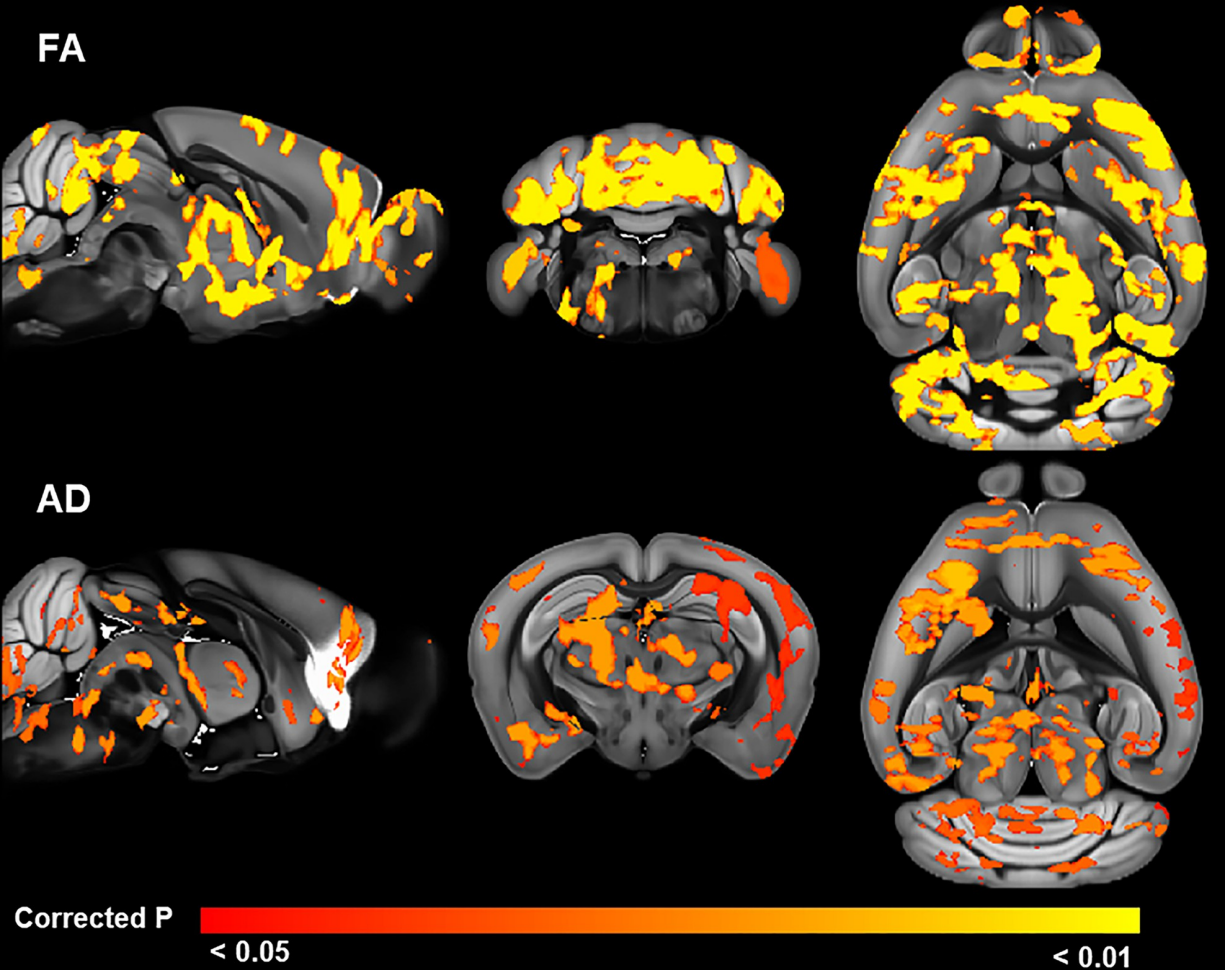
Relationship between EE and brain microstructure in the juvenile cohort. Brain regions (red to yellow areas) with significantly (TFCE and FWE corrected p < 0.05) increased FA (top) or AD (bottom) after EE.

For the juvenile cohort, there was a significant (TFCE and FWE corrected p < 0.05) main effect of housing on both FA and AD in predominantly gray matter areas throughout the brain indicating widespread microstructural changes associated with EE. There were no significant sex main effects or housing by sex interactions on FA or AD. There were no significant main housing, sex, or housing by sex interaction effects for MD or RD. Post hoc t-tests revealed regions with increased FA in EE mice including clusters with signal peaks in the entorhinal cortex, hippocampus, cerebellum, brainstem reticular formation and sensory nuclei/white matter tracts, superior and inferior colliculi, basal forebrain, corpus callosum, thalamus, striatum, and sensory, limbic, and motor cortical regions (Fig 8; S15 Table in S1 Appendix). Post hoc t-tests revealed increased AD in EE mice in the hypothalamus, ventral tegmental area, cerebellum, entorhinal cortex, hippocampus and alveus, striatum, insula, cortical and subcortical motor areas, and sensory cortical regions (Fig 8; S16 Table in S1 Appendix).

#### Repetitive motor behavior associated with lower FA in juvenile C58 mice

**Fig 9:**
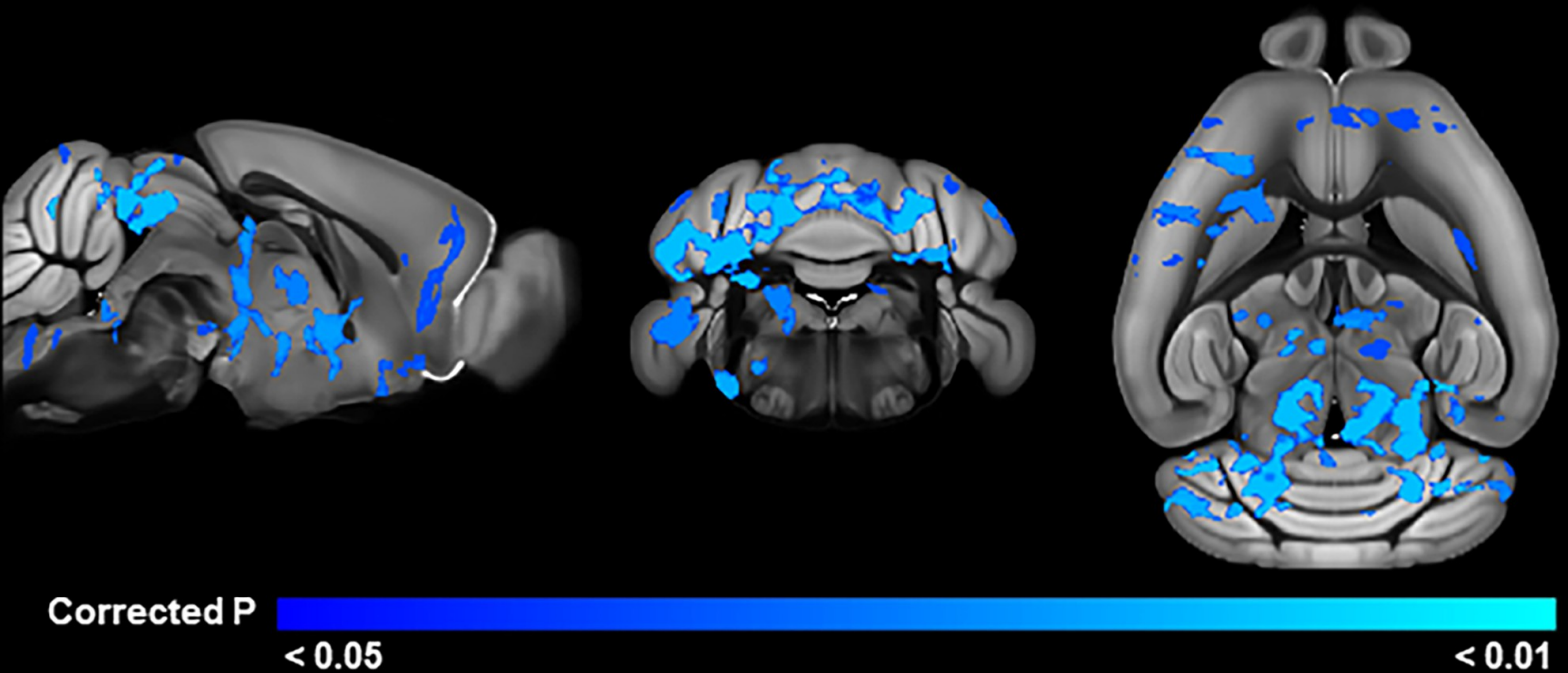
Brain regions with negative correlations between FA and RRB in the juvenile cohort. Blue areas indicate regions with significant (TFCE and FWE corrected p < 0.05) correlations.

In the juvenile animals, there was a significant (TFCE and FWE corrected p < 0.05) negative correlation between repetitive motor behavior scores and FA (higher repetitive motor behavior was associated with lower FA) in a number of gray matter regions throughout the brain, as well as some white matter tracts (Fig 9; S17 Table in S1 Appendix). These significant regions included clusters with signal peaks in cortical and subcortical motor and sensory processing regions; the entorhinal cortex and hippocampal regions; reward centers including the nucleus accumbens; central amygdala nucleus and other regions associated with fear and limbic processing; autonomic hypothalamic regions; thalamic regions associated with attention, pain and somatosensory processing; as well as multiple regions of the corpus callosum and multiple nuclei within the reticular formation. Post hoc Spearman rank correlations revealed that the brain regions with the strongest negative correlations (r > 0.8) between FA and repetitive motor behavior were in the right cerebellar simple lobule, right nucleus accumbens, corpus callosum anterior forceps, cerebellum lobules IV-V, and right spinal nucleus of the trigeminal nerve. There were no areas in which a positive correlation between FA and repetitive motor behavior was observed. There were no significant positive or negative correlations between MD, AD, or RD and repetitive motor behavior in any brain region.

#### EE increases FA and AD in RRB-associated regions in juvenile C58 mice

**Fig 10:**
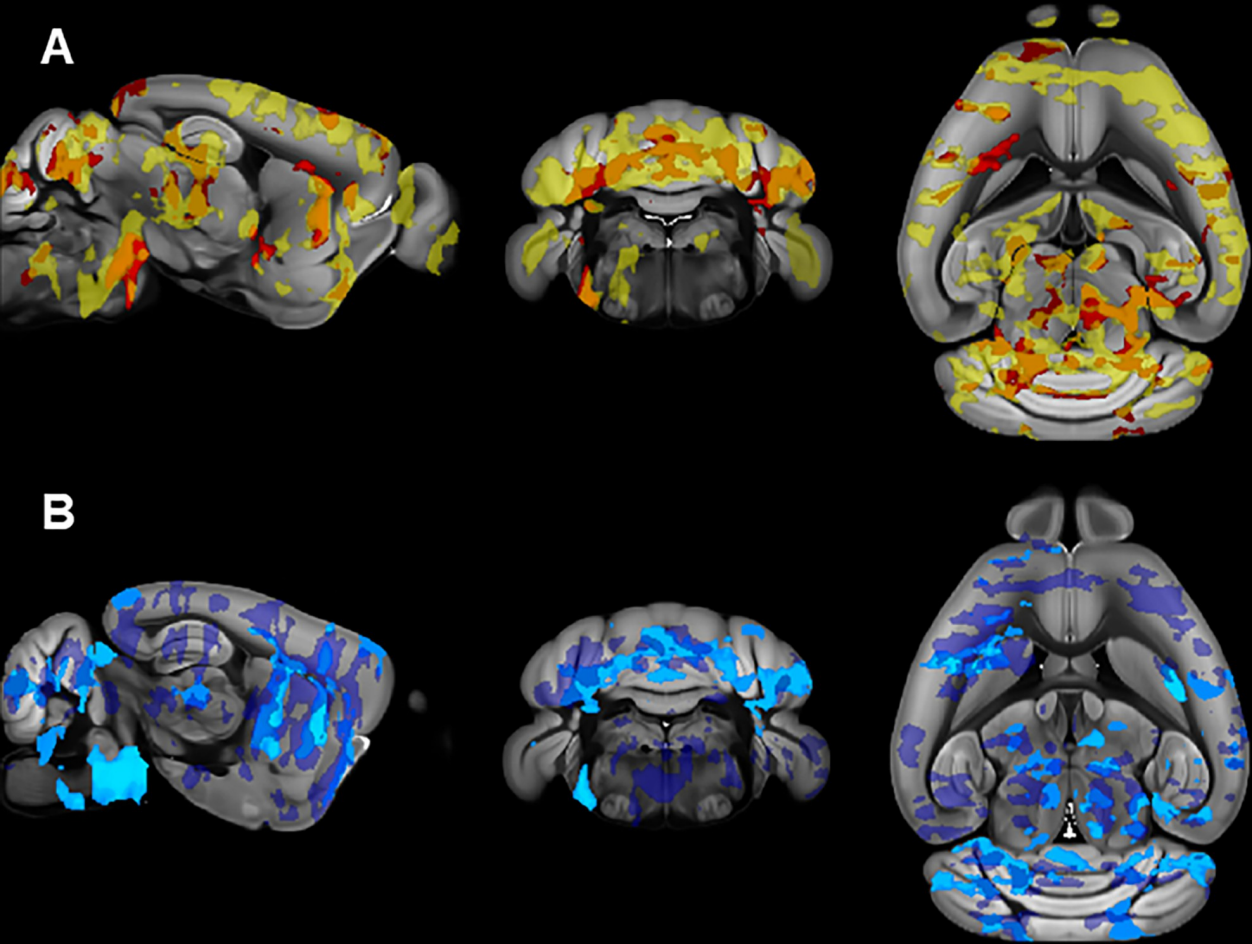
Overlap between EE-induced microstructural differences and regions with RRB correlations in juvenile mice. A) Overlap (orange) between brain regions with EE-induced increases in FA (yellow) and negative correlations between RRB and FA (in red). B) Overlap (medium blue) between brain regions with EE-induced increases in AD (dark blue) and negative correlations between RRB and FA (light blue).

**Fig 11:**
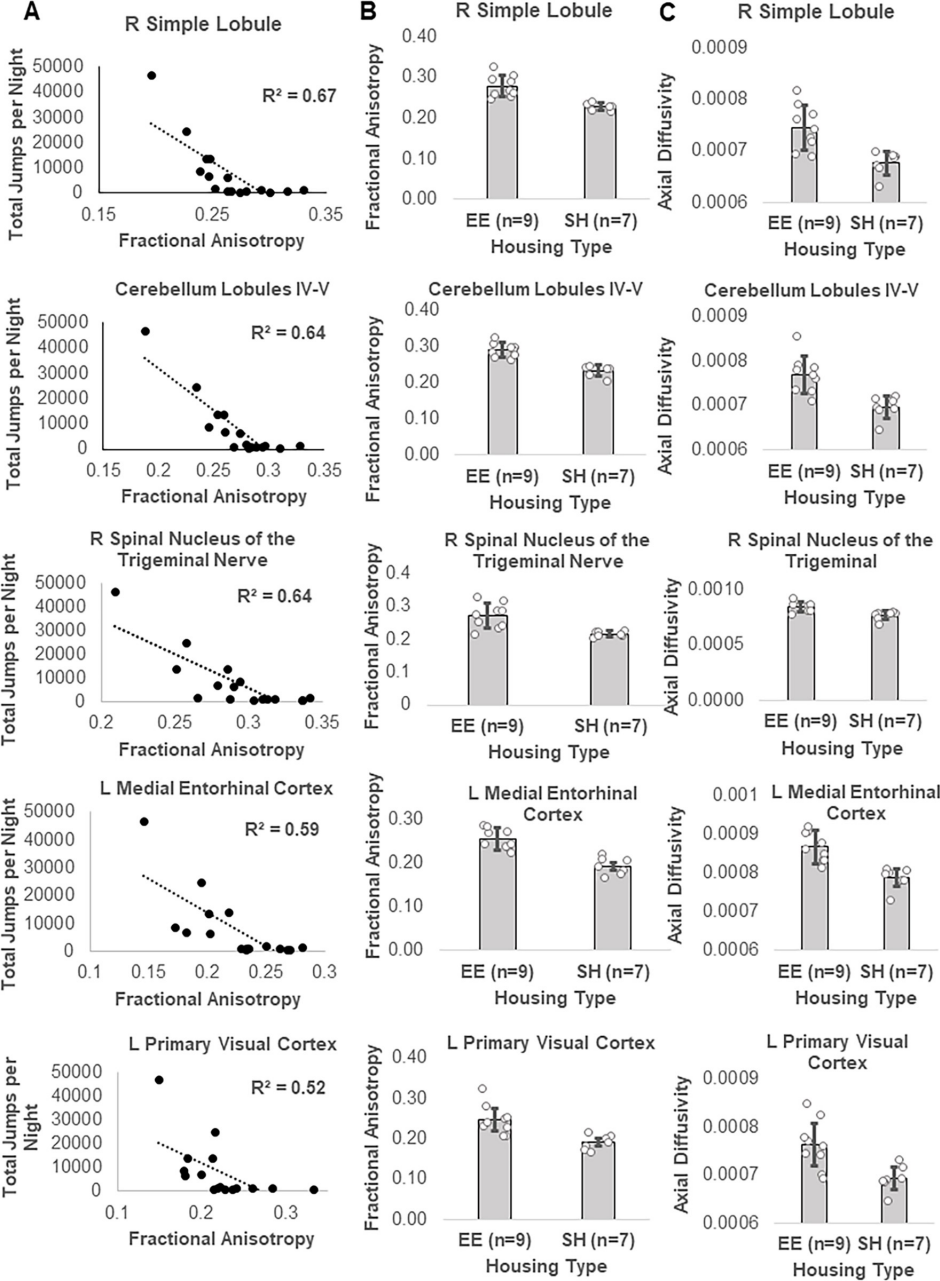
Relationship between EE-induced alterations in FA and AD and RRB-FA correlations in juvenile C58 mice. A) Correlation between RRB and FA by brain region. B) FA differences between housing groups by brain region. C) AD differences between housing groups by brain region.

Several regions exhibited overlap between EE-associated increases in FA and AD and negative correlations between FA and repetitive motor behavior (Figs 10 and 11). These overlapping areas may indicate brain regions where EE induced microstructural changes that reduced RRB expression. Regions with very strong correlations (r > 0.7) between RRB and FA and overlap with EE-induced increases in both FA and AD included the cerebellar right simple lobule, arbor vitae, and left primary visual area. Overlapping areas in both FA and AD with strong RRB correlations with FA (r > 0.5) included the right superior colliculus motor-related regions. Overlapping areas in both FA and AD with moderate RRB correlations with FA (r > 0.3) included the left and right striatum and right supplemental somatosensory area. Regions of overlap between only EE increases in FA and regions with RRB correlations with FA included very strong RRB correlations with cerebellar lobules IV-V, right spinal trigeminal nucleus, and left dorsal part of the medial entorhinal cortex; strong correlations with the left central lateral thalamic nucleus, right inferior cerebellar peduncle, left cerebellum interposed nucleus, right primary somatosensory area, right medial vestibular nucleus, right ventral cochlear nucleus, and left hippocampus dentate gyrus molecular layer, CA1, and CA3; and moderate correlations with the corpus callosum body, left lateral posterior thalamic nucleus, right midbrain reticular nucleus, right pontine reticular nucleus, and right visceral area.

## Discussion

Relatively few studies have used DTI to assess the relationship between brain microstructure and RRB [32–35] even though this technique has been widely used to identify biomarkers of pathology in other disease models. This is the first DTI study to explore how an enriched environment can modify brain microstructure to reduce the development of RRB in an animal model. In this study, we examined the effect of EE on the C58 mouse model of RRB in two age cohorts compared with control mice (C57) that do not exhibit RRB. This study sought to address the questions of 1) how brain microstructure is altered in C58 mice exhibiting high levels of RRB, 2) how RRB-associated microstructural differences change over the course of development, and 3) how brain microstructure is influenced by EE to attenuate the expression of RRB in C58 mice.

### Effects of EE on repetitive behavior

As expected, SH C58 mice exhibited much higher rates of RRB than did similarly housed C57 mice. Interestingly, only SH C58 female mice had significantly higher rates of RRB than same-sex SH C57 mice. Also as expected, EE markedly attenuated the expression of RRB in both age cohorts of C58 mice. For both age cohorts, EE significantly reduced RRB in female, but not male, C58 mice due to low RRB in some SH C58 male mice.

### Strain differences in brain microstructure

When strain differences in brain microstructure were examined between C58 mice and the C57 control strain, widespread differences were observed in the adult cohort, with C58 mice exhibiting reduced values for all diffusion metrics in regions throughout the brain. Furthermore, strain differences in FA and RD, particularly in the right dorsomedial and caudal striatum, appeared to be strongly associated with RRB in this age cohort. Wilkes et al. [32] examined diffusion metric differences between C58 and C57 mice housed under standard conditions in the major white matter tracts of the cerebellum and basal ganglia and found reduced FA and AD in the inferior cerebellar peduncle (ICP) of C58 mice compared to C57 mice when both strains were housed under standard conditions. However, they did not find any correlations between RRB and diffusion metrics. Interestingly, we did not find strain differences in FA or AD in the ICP in our adult cohort but did find housing effects and strong RRB associations with FA in the right ICP in the juvenile cohort. Differences in results between our study and Wilkes et al. [32] may be reflective of methodological differences, as their work was performed *ex vivo* whereas the present studies were performed *in vivo*. Moreover, the statistical models in this study also included the factor of housing condition and our brain-wide approach necessitates many more comparisons than their region-of-interest approach, which targeted a smaller number of white matter tracts resulting in lower statistical power.

### EE effects on brain microstructure

Our results suggest that EE has profound effects on brain microstructure, particularly in juvenile C58 mice. In the juvenile cohort of the development study, EE C58 mice exhibited increased FA and AD in regions throughout the brain, predominantly in gray matter. Many of these regions with increased FA and AD overlapped. Multiple cerebellar regions exhibited increased FA and AD in juvenile EE C58 mice including regions associated with motor function (simple lobule, copula pyramidis) [49, 50], eye movements (nodulus) [51], and higher level cognitive functions (crus II) [49]. Outside the cerebellum, EE-induced increases in both FA and AD were observed in brain regions associated with movement (superior colliculus), sensory processing (olfactory tubercle, piriform cortex, primary visual cortex, supplemental somatosensory area), learning and memory (hippocampus CA1), spatial navigation (medial entorhinal cortex), and action selection (striatum). Many of these same regions also exhibited strong negative correlations between FA and repetitive motor behavior, particularly in cerebellar and sensory processing regions. This overlap suggests that EE may attenuate RRB by microstructural changes in these regions.

The effects of housing condition on brain microstructure was less clear in the adult cohort and appeared to be sex and strain dependent. Female mice housed in EE exhibited reduced FA in the left agranular insular, primary motor, and primary somatosensory cortices. Interestingly, there were no significant differences in FA between C58 EE and SH female mice despite a large reduction in repetitive motor behavior in EE C58 female mice in this older cohort. In addition, RRB exhibited a negative association with FA in the adult cohort, which suggests there is not a direct relationship between EE-induced FA decreases in female mice and the attenuation of RRB in EE C58 mice in the older cohort. This may indicate that housing effects on RRB development predominantly occur in the earlier 3-week postweaning timepoint before RRB is fully established. Alternatively, it could suggest that EE-induced FA decreases in female mice in the insular cortex and sensorimotor cortices indirectly reduce RRB through interactions with other brain regions.

Considering EE effects in both age cohorts, our hypothesis that EE attenuates RRB by inducing microstructural alterations in the indirect pathway of the basal ganglia nuclei and cerebellum, is partially supported. Overlap between EE-induced increases in FA and strong negative correlations between RRB and FA in several cerebellar regions (right simple lobule, cerebellar lobules IV-V, arbor vitae, right inferior cerebellar peduncle, left cerebellum interposed nucleus) in the younger cohort provides strong support of the role of EE-induced cerebellar changes in the attenuation of RRB. In contrast, while we found evidence of basal ganglia regions (striatum) mediating EE-induced attenuation of RRB, we did not find evidence for a strong role of the indirect basal ganglia pathway as we did not observe overlap between EE-induced effects and RRB associations in basal ganglia regions unique to the indirect pathway. This lack of support for the indirect basal ganglia pathway in EE-induced attenuation of RRB is surprising given past studies that show associations between reduced RRB and EE- induced alterations in dendritic complexity in basal ganglia nuclei, particularly the subthalamic nucleus [15, 16, 18]. It should be noted, though, that alterations of cerebellar regions would be expected to affect basal ganglia function given their strong functional and structural connections [52] and that EE-induced alterations of the cerebellum in the younger cohort would be expected to alter the microstructure of the left and right striatum, which were strongly associated with RRB in the adult cohort.

Decreased FA is commonly observed in instances of white and gray matter pathology but is relatively nonspecific as to the exact cause [20, 21]. Decreased AD in white matter is commonly interpreted as an indicator of axonal loss or poor axonal integrity, but in gray matter can also indicate altered, typically increased, dendritic density [21, 53]. EE has been commonly associated with synaptogenesis and increased dendritic complexity [54, 55]. Lewis et al. [15] observed increased dendritic spine density in the subthalamic nucleus after EE in C58 mice. Thus, the increased FA and AD observed in the juvenile EE C58 mice may reflect altered dendritic complexity in gray matter regions. It should be noted, however, that most authors consider the combination of increased FA and AD values to generally indicate a decrease in dendritic complexity that would not be expected under EE conditions [53, 56]. In contrast, Reveley et al. [29] found that the large abundance of apical dendrites of layer 5 pyramidal neurons in the marmoset cerebral cortex have a vertical orientation, which resulted in high FA and AD values. In white matter regions, the increased FA and AD we observed in response to EE may reflect increased axonal density. The overlap of brain regions between the EE-induced increases in FA and AD in the juvenile cohort and the brain regions with negative correlations between FA and RRB suggest that RRB was reduced by EE using the same neurocellular processes responsible for the widespread EE-induced changes in FA and AD. However, this is not clearcut as no correlation between RRB and AD was found. Negative correlations between RRB and FA and positive correlations with RD measures in the adult cohort may also reflect associations between RRB and neurite density in gray matter regions or could reflect the influence of another process such as a negative association between RRB and the amount of myelination along axons in both white and gray matter [21, 29, 53]. Increased RD has commonly been linked to myelin decreases in white matter tracts but its interpretation in gray matter is less straightforward [21]. Additional histological studies are necessary to confirm the cause of the diffusion metric differences observed in this study. Nevertheless, the diffusion metric differences observed in this study are indicators of pathology in the identified brain regions. The identification of altered brain regions in this study can be used to guide further studies of the neurocircuitry of RRB, as well as direct the search for the specific neurocellular processes underlying RRB and its attenuation by EE.

### Associations between RRB and brain microstructure

Our results support the notion that the relationship between repetitive motor behavior and regional brain microstructure changes over the course of development. We observed correlations between RRB and diffusion measures in different brain regions in the two age cohorts. Juvenile mice displayed the strongest correlations between RRB and FA in predominantly gray matter cerebellar and sensory processing regions, whereas young adult mice displayed correlations between RRB and FA and RD in the striatum and white matter tracts. This may reflect the result of early cerebellar dysfunction affecting the development of striatal function, as well as alterations in myelination of white matter tracts due to changes in neuronal activation. The cerebellum and basal ganglia are interconnected by multiple pathways and are functionally integrated such that dysfunction in one area is theorized to lead to dysfunction or compensatory functioning in the other area [52]. Our findings are similar to those of Wolff et al. [35], who observed associations between FA in cerebellar and corpus callosum white matter tracts in human infants at high risk for autism with measures of RRB at age 2, although they observed a positive association between FA and RRB measures. They similarly speculated that autism symptoms, including RRB, in early development may be related cerebellar dysfunction that may drive the striatal differences commonly seen in older children and adults with autism. Recent evidence suggests that myelination of white matter tracts is plastic and responsive to levels of neuronal activation–thus alterations in gray matter microstructure and resulting functional changes in our younger cohort may have altered myelination and corresponding RD values in our adult cohort [57, 58].

Cerebellar dysfunction has been widely reported in autism and is one of the brain regions most commonly associated, usually as volumetric differences, with RRB in MRI studies [36, 59]. For instance, human studies have shown negative correlations between RRB and the volume of cerebellar lobules IV-V, VIIIB, simple lobule, and crus I and II, as well as positive correlations between the volume of vermis lobules VIIB and VIIIA and RRB [60, 61]. In a longitudinal study in young children, Wolff et al. [35] found that Repetitive Behavior Scale-Revised (RBS-R) scores at age 2 were consistently positively associated with FA in the middle cerebellar peduncle and superior cerebellar peduncle, as well as the corpus callosum genu from 6 to 24 months of age. Using an animal model, Stoodley et al. [59] showed that inhibition of Purkinje neurons in the right crus I of the cerebellum resulted in repetitive grooming and cognitive inflexibility in a reversal learning task. We believe that our findings lend further support to the conceptual framework that cerebellar changes in early development can influence the trajectory of RRB.

The strong correlations between RRB and FA in sensory processing regions in the juvenile cohort raise interesting questions about the origins of RRB. Sensory processing anomalies including hypersensitivity have been commonly reported in ASD and appear to be correlated with rates of RRB [62, 63]. Furthermore, Wang et al. [64] suggested that RRB was the result of impaired sensory habituation as Sprague-Dawley rats raised under impoverished conditions displayed both reduced habituation to a light stimulus and increased RRB compared to animals raised under enriched conditions. Lee et al. [65] demonstrated that combining sensory (auditory) stimuli with optogenetic excitation or inhibition of the cerebellum simple lobule increased limb movement and led to sensorimotor learning, which paired the movement with the sensory stimulus. The particularly strong correlations between RRB and microstructure in the right simple lobule and sensory processing regions could be similarly interpreted to suggest that altered sensory processing could result in RRB through deficits in habituation or aberrant sensorimotor learning. Future studies should explore whether the association of RRB with altered microstructure in cerebellar and sensory processing regions is causative.

Our data also suggest there may be an association between RRB and brainstem movement circuits, as we observed strong correlations with RRB in parts of the reticular formation such as the parvicellular reticular nucleus, pedunculopontine nucleus, and caudal part of the pontine reticular nucleus. In addition to roles in sensorimotor gating, which is relevant to sensory processing and habituation, nuclei within the reticular formation also participate in circuits governing movement. In a recent paper, Inagaki et al. [66] described a circuit from the midbrain reticular and pedunculopontine nuclei to the motor cortex via the thalamus, which serves as a signal to initiate a planned motor command. Interestingly, this part of the thalamus also receives input from the basal ganglia, superior colliculus, and cerebellum [66]. Nuclei within the reticular formation also project to central pattern generators in the spinal cord through the reticulospinal tract [67], which could similarly be relevant to the development of motor stereotypies.

Our results also suggest a potential role for aberrant memory encoding in the medial entorhinal cortex in RRB. The strong negative correlation between RRB and FA in the medial entorhinal cortex (MEC) and its altered microstructure following EE in the juvenile cohort suggest this region is important for both neural dysfunction in RRB and the attenuation of RRB by EE. Previous studies have associated this region with RRB, including increased jumping behavior [68–70], as well as alterations following EE [71–74]. Earlier studies suggest that stereotypy results from increased dopamine in the nucleus accumbens and amygdala following MEC lesions [69, 70, 75]. Scholz et al. [73] observed increased entorhinal cortical volume and improved spatial learning in a Barnes maze in male C57BL/B6 mice following 3 weeks of EE exposure. The MEC serves as a bidirectional gateway between the cortex and hippocampus that integrates sensory and other cortical information with spatial context to aid in learning and memory formation [76, 77]. This brain region plays key roles in spatial representation, navigation, and memory as well as episodic and associative memory and the temporal coordination of hippocampal neuronal activity [78–80]. Thus, microstructural alterations in this region have the potential to cause RRB through alterations of dopamine in limbic regions and may also negatively impact the development of other brain regions by projection of aberrant spatial associations to the cortex and disruption of hippocampal firing patterns.

### Study limitations

Although this study has identified brain regions and microstructural changes associated with RRB and EE’s attenuation of RRB, it has several important limitations. Partial volume effects, which can occur when multiple tissue types are contained within a single voxel and their signal intensities are averaged, may have influenced the results observed here, particularly along the edge of the ventricles and at other tissue boundaries [81]. Partial volume effects in diffusion studies are of particular concern for gray matter, which has tissue-specific diffusion properties [81]. This study also lacked a strain control group in younger cohort, which would have aided in separating the effects of EE on RRB from strain-specific EE effects. Because the focus of this study centers on RRB, we chose to focus on C58 mice in our developmental study to better understand the neurocircuitry underlying the development of RRB and its attenuation by EE, whereas juvenile C57 mice show near-zero levels of stereotyped motor behavior at 3-weeks post weening [9]. Thus, while our developmental study cannot inform as to whether juvenile C57 and C58 are affected similarly by EE, it does contribute to our understanding of how EE alters the brains and behavior of C58 mice during development. It is also possible that MRI scans earlier in development might have provided additional insights about the development of RRB in both housing treatments. MRI scans at earlier developmental timepoints were probably not logistically feasible, however, given the small size of mice at such an early age. A longitudinal study design would have also been beneficial to reduce individual variation across time and allowed for the tracking of housing-induced changes in the brains and behavior of individuals over time. It would be beneficial for a longitudinal study to be conducted to see if the results of this study can be replicated. A crossover study would also be useful to observe changes in brain-behavior relationships before and after each housing treatment, as well as to investigate how the brain responds to the cessation of EE.

## Conclusions

**Fig 12:**
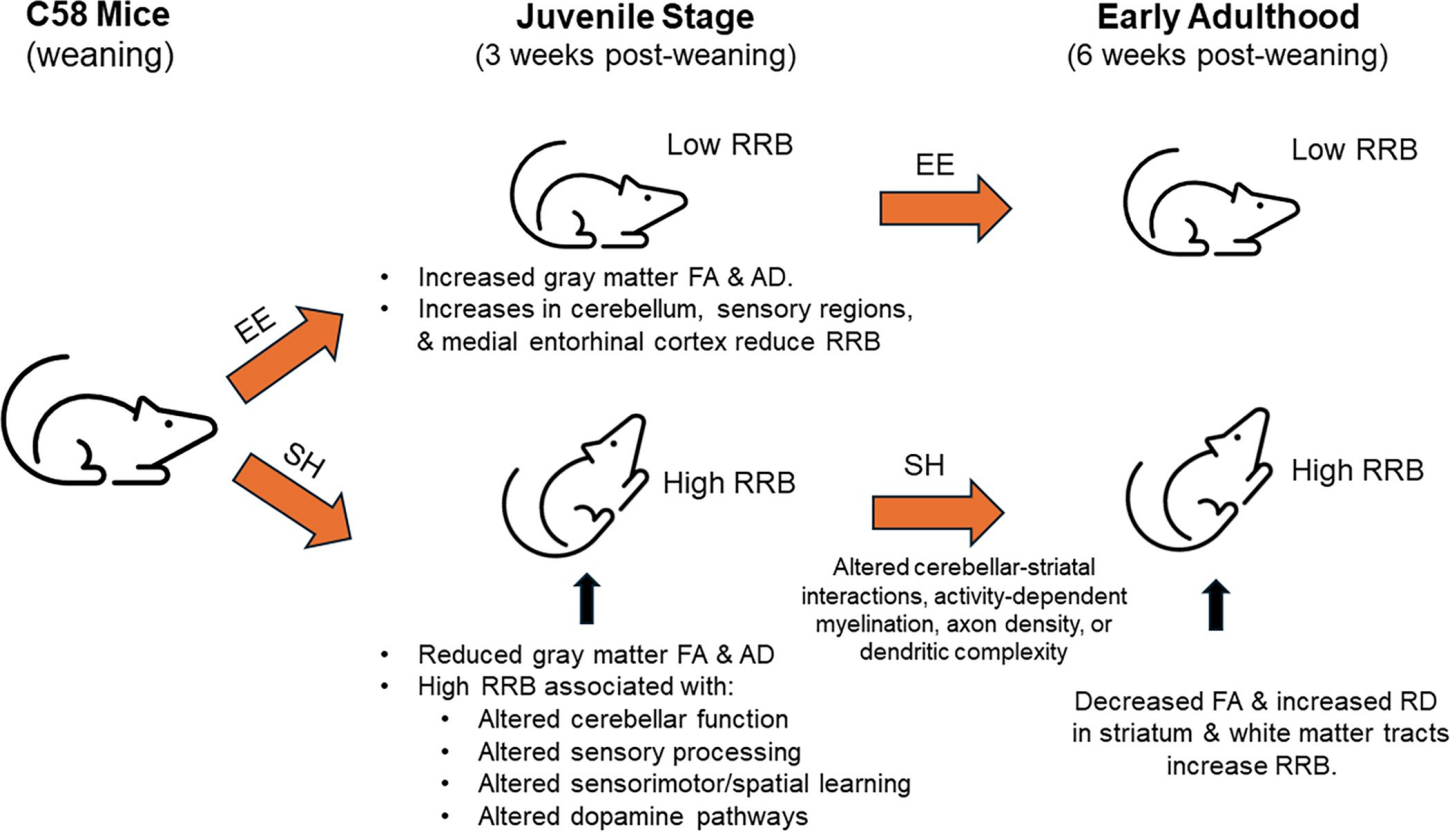
Conceptual model of how EE attenuates RRB in C58 mice.

EE-induced widespread changes in brain microstructure, including increased FA and AD, in the juvenile mice of our development study. A subset of these brain regions showed strong negative correlations between FA and RRB, suggesting these regions are both associated with RRB development and its attenuation by EE. These regions included cerebellar regions important in movement and motor learning, sensory processing areas, and a brain region involved in spatial navigation and memory. In our adult study, widespread strain differences were observed in all diffusion metrics indicating brain-wide microstructural alterations in the C58 inbred mouse strain. A subset of these regions in the left and right striatum, corpus callosum body and genu, internal and external capsules, were correlated with RRB, suggesting that RRB in the adult cohort is associated with strain-related microstructural differences. Our results further suggest that adolescent sensory and cerebellar dysfunction in conjunction with dysfunction in the medial entorhinal cortex play a role in the development of RRB in C58 mice, and that dysfunction in these regions changes the trajectory of brain development in the basal ganglia and white matter tracts in adult C58 mice. Contrary to our initial hypothesis, EE appears to reduce RRB by altering the microstructure of regions throughout the brain in juvenile animals, preventing the decreased FA in the medial entorhinal cortex and cerebellar and sensory regions associated with RRB in adolescence and leading to further microstructural alterations in striatal regions and white matter tracts in adulthood (Fig 12). This study suggests a fundamental rather than peripheral role for sensory processing deficits in the development of RRB, which should be further explored.

## Supporting information

S1 Appendix (DOCX)

S1 Data (XLSX)
