# Supplementary material for: Environmental enrichment reduces restricted repetitive behavior by altering gray matter microstructure
Source: PLoS One. 2024 Jul 31;19(7):e0307290. doi: 10.1371/journal.pone.0307290 (PMC11290697; doi:10.1371/journal.pone.0307290)
Supplement: S1 Appendix — (DOCX) [file pone.0307290.s001.docx]

**Table S1.** Clusters with significantly lower FA in adult C58 mice.

| Diffusion Metric | Cluster  No. | Cluster Voxels | Location (Hemisphere) | T-statistic |
| --- | --- | --- | --- | --- |
| FA | 119 | 1151824 | Optic tract (R) | 10.50 |
|  |  |  | Striatum (R) | 10.10 |
|  |  |  | Lateral ventricle (R) | 9.90 |
|  |  |  | Stria terminalis (R) | 9.89 |
|  |  |  | Bed nucleus of the stria terminalis (R) | 9.79 |
|  |  |  | Dorsomedial striatum (L) | 9.54 |
|  | 118 | 6476 | Primary somatosensory area – upper limb (L) | 3.37 |
|  |  |  | Primary somatosensory area – barrel field (L) | 3.29 |
|  |  |  | Primary somatosensory area – nose layer (L) | 2.99 |
|  |  |  | Primary somatosensory area – unassigned (L) | 2.98 |
|  | 117 | 1284 | Retrosplenial area lateral agranular part (R) | 4.39 |
|  |  |  | Primary somatosensory area – lower limb (R) | 4.27 |
|  |  |  | Primary somatosensory area – upper limb (R) | 3.63 |
|  | 116 | 1148 | Anteromedial thalamic nucleus – dorsal part (R) | 3.07 |
|  |  |  | Ventral anterior-lateral thalamic complex (R) | 2.69 |
|  |  |  | Paracentral thalamic nucleus (R) | 2.26 |
|  |  |  | Mediodorsal thalamic nucleus (R) | 2.22 |
|  |  |  | Stria medullaris (R) | 1.78 |
|  | 115 | 717 | Main olfactory bulb (R) | 3.61 |
|  | 114 | 502 | Olfactory areas (R) | 2.76 |
|  | 113 | 383 | Medial mammillary nucleus – medial part (L) | 2.85 |
|  |  |  | Periventricular hypothalamic nucleus – posterior part (L) | 2.69 |
|  | 112 | 247 | Primary visual area (L) | 2.73 |
|  | 111 | 226 | Spinal nucleus of the trigeminal nerve (R) | 3.48 |
|  | 110 | 137 | Superior colliculus optic layer (L) | 2.09 |
|  | 109 | 136 | Pallidum (R) | 2.91 |
|  | 108 | 118 | External capsule (R) | 2.18 |
|  | 107 | 116 | Presubiculum (L) | 2.35 |
|  |  |  | Postsubiculum (L) | 1.72 |
|  | 106 | 102 | Supplemental somatosensory area (L) | 2.09 |
|  | 105 | 83 | Piriform area (R) | 2.57 |
|  | 104 | 80 | Principal sensory nucleus of the trigeminal nerve (L) | 2.14 |
|  | 103 | 72 | Olfactory nerve layer of olfactory bulb (R) | 2.08 |
|  | 101 | 55 | Secondary motor area (L) | 2.30 |
|  |  |  | Agranular insular area - dorsal part (L) | 1.93 |
|  | 99 | 52 | Arbor vitae | 2.16 |
|  | 98 | 46 | Reticular thalamic nucleus (R) | 2.31 |
|  | 97 | 42 | Triangular nucleus of septum (R) | 2.10 |
|  | 96 | 42 | Retrosplenial area dorsal part (L) | 2.19 |
|  |  |  | Retrosplenial area lateral agranular part (L) | 2.13 |
|  | 95 | 37 | Pontine reticular nucleus caudal part (R) | 2.04 |
|  | 93 | 36 | Anterior olfactory nucleus (R) | 1.92 |
|  | 92 | 35 | Posteromedial visual area (L) | 2.03 |
|  | 91 | 34 | Main olfactory bulb (L) | 1.89 |
|  | 90 | 33 | Superior vestibular nucleus (L) | 1.97 |
|  | 88 | 30 | Secondary motor area (L) | 1.83 |
|  | 86 | 21 | Hippocampus CA1 (L) | 1.82 |
|  | 85 | 21 | Primary auditory area (L) | 2.66 |
|  | 84 | 19 | Superior colliculus motor related (L) | 1.83 |
|  | 83 | 19 | Cerebellum lobule II | 1.86 |
|  | 82 | 19 | Ventral auditory area (R) | 1.84 |
|  | 80 | 13 | Cerebellum lobule III | 1.84 |
|  | 79 | 12 | Substantia nigra reticular part (R) | 1.91 |
|  | 78 | 12 | Prosubiculum (R) | 1.80 |
|  | 75 | 10 | Lateral olfactory tract body | 2.04 |
|  | 74 | 9 | Secondary motor area (L) | 1.76 |
|  | 73 | 9 | Taenia tecta ventral part (R) | 1.84 |
|  | 69 | 7 | Midbrain (R) | 1.85 |
|  | 67 | 5 | Taenia tecta ventral part (L) | 1.86 |
|  | 66 | 5 | Agranular insular area posterior part (R) | 1.71 |
|  | 63 | 4 | Entorhinal area lateral part (L) | 1.74 |
|  | 62 | 4 | Pons (L) | 1.76 |
|  | 61 | 4 | Rostrolateral visual area (L) | 1.98 |
|  | 60 | 3 | Entorhinal area lateral part (R) | 1.85 |
|  | 57 | 3 | Cerebellum declive | 1.96 |
|  | 56 | 3 | Sensory root of the trigeminal nerve (L) | 1.97 |
|  | 55 | 3 | Perirhinal area (R) | 1.80 |
|  | 54 | 2 | Primary motor area (L) | 1.70 |
|  | 53 | 2 | Anterior visual area (L) | 1.68 |
|  | 52 | 2 | Primary visual area (L) | 1.71 |
|  | 49 | 2 | Fimbria (L) | 1.74 |
|  | 48 | 2 | Retrosplenial area ventral part (R) | 1.73 |
|  | 46 | 2 | Supplemental somatosensory area (L) | 1.68 |
|  | 45 | 2 | Presubiculum (L) | 1.69 |
|  | 44 | 2 | Ventral auditory area (R) | 1.85 |
|  | 37 | 1 | Gigantocellular reticular nucleus (R) | 1.67 |
|  | 36 | 1 | Rostrolateral visual area (R) | 1.67 |
|  | 34 | 1 | Entorhinal area medial part dorsal (R) | 1.69 |
|  | 32 | 1 | Cortical amygdala area posterior part (L) | 1.68 |
|  | 29 | 1 | Olfactory tubercle (R) | 1.78 |
|  | 28 | 1 | Lateral olfactory tract (L) | 1.76 |
|  | 26 | 1 | Visual anterior area (L) | 1.70 |
|  | 24 | 1 | Olfactory areas (L) | 1.68 |
|  | 23 | 1 | Olfactory tubercle (L) | 1.71 |
|  | 20 | 1 | Hippocampus CA1 (L) | 1.68 |
|  | 19 | 1 | Dentate gyrus molecular layer (L) | 1.69 |
|  | 18 | 1 | Dorsal auditory area (R) | 1.82 |
|  | 15 | 1 | Primary motor area (R) | 1.69 |
|  | 14 | 1 | Globus pallidus external segment (L) | 1.67 |
|  | 12 | 1 | Cerebellum simple lobule (R) | 1.72 |
|  | 11 | 1 | Secondary motor area (R) | 1.70 |
|  | 9 | 1 | Supplemental somatosensory area (R) | 1.60 |
|  | 7 | 1 | Superior colliculus zonal layer (R) | 1.68 |
|  | 5 | 1 | Ventral posteromedial thalamic nucleus (R) | 1.67 |

**Table S1 Legend:** Cluster number, cluster size in voxels, and brain regions with peak t-values for clusters with significantly lower (TFCE and FWE-corrected p < 0.05) fractional anisotropy (FA) in C58 mice compared to C57 mice based on a post hoc t-test conducted in FSL randomise. Cluster information extracted using the FSL Cluster tool. Clusters with maxima at duplicate regions not shown. FWE = familywise error rate. TFCE = threshold-free cluster enhancement.

**Table S2**. Clusters with significantly lower AD in adult C58 mice.

| Diffusion Metric | Cluster  No. | Cluster Voxels | Location (Hemisphere) | T-statistic |
| --- | --- | --- | --- | --- |
| AD | 39 | 2468445 | Periventricular hypothalamic nucleus -preoptic part (L) | 14.00 |
|  |  |  | Periventricular hypothalamic nucleus – intermediate part (R) | 11.90 |
|  |  |  | Arcuate hypothalamic nucleus (L) | 11.80 |
|  |  |  | Arbor vitae | 9.82 |
|  |  |  | Dentate gyrus molecular layer (L) | 9.81 |
|  | 38 | 264 | Olfactory areas (R) | 2.28 |
|  | 37 | 92 | Corpus callosum genu | 2.38 |
|  | 36 | 45 | Cerebellum crus I (L) | 2.91 |
|  | 34 | 31 | Medial amygdala nucleus (R) | 2.59 |
|  | 33 | 24 | Primary motor area (R) | 1.92 |
|  | 32 | 23 | Spinal tract of the trigeminal (L) | 1.85 |
|  | 31 | 16 | Visual rostrolateral area (L) | 2.61 |
|  | 30 | 15 | Temporal association areas (R) | 1.87 |
|  | 29 | 13 | Cerebellum crus II (R) | 2.41 |
|  | 27 | 7 | Olfactory nerve layer of olfactory bulb (L) | 1.87 |
|  | 26 | 7 | Anterolateral visual area (R) | 1.97 |
|  | 24 | 4 | Primary somatosensory area -mouth (R) | 1.74 |
|  | 23 | 3 | Cerebellum paramedian lobule (R) | 2.52 |
|  | 20 | 2 | Retrosplenial area ventral part (R) | 1.69 |
|  | 19 | 2 | Piriform area (L) | 1.84 |
|  | 18 | 2 | Dentate gyrus molecular layer (R) | 1.82 |
|  | 17 | 2 | Main olfactory bulb (L) | 1.78 |
|  | 16 | 2 | Tuberal nucleus (R) | 1.72 |
|  | 14 | 2 | Olfactory areas (L) | 1.69 |
|  | 13 | 2 | Pontine reticular nucleus (R) | 1.68 |
|  | 12 | 2 | Main olfactory bulb (R) | 1.71 |
|  | 11 | 1 | Cerebellum lobules IV-V (L) | 1.69 |
|  | 10 | 1 | Cortical amygdala area posterior part (L) | 1.75 |
|  | 9 | 1 | Secondary motor area (R) | 1.68 |
|  | 8 | 1 | Primary somatosensory area – barrel field (R) | 1.79 |
|  | 7 | 1 | Retrochiasmic area (R) | 1.68 |
|  | 6 | 1 | Primary somatosensory area – barrel field (L) | 1.69 |
|  | 3 | 1 | Ectorhinal area (R) | 1.71 |
|  | 2 | 1 | Anterior visual area (L) | 1.70 |

**Table S2 Legend**: Cluster number, cluster size in voxels, and brain regions with peak t-values for clusters with significantly (TFCE and FWE-corrected p < 0.05) lower axial diffusivity (AD) in C58 mice compared to C57 mice based on a post hoc t-test conducted in FSL randomise. Cluster information extracted using the FSL Cluster tool. Clusters with maxima at duplicate regions not shown. FWE = familywise error rate. TFCE = threshold-free cluster enhancement.

**Table S3**. Clusters with significantly lower RD in adult C58 mice.

| Diffusion Metric | Cluster  No. | Cluster Voxels | Location (Hemisphere) | T-statistic |
| --- | --- | --- | --- | --- |
| RD | 45 | 2142998 | Arcuate hypothalamic nucleus (L) | 12.90 |
|  |  |  | Periventricular hypothalamic nucleus – preoptic part (L) | 11.30 |
|  |  |  | Cerebellum simple lobule (L) | 11.20 |
|  |  |  | Arcuate hypothalamic nucleus (R) | 10.90 |
|  |  |  | Cerebellum flocculus (L) | 10.90 |
|  | 44 | 438 | Medial vestibular nucleus (R) | 3.03 |
|  | 43 | 88 | Lateral lemniscus (L) | 2.16 |
|  |  |  | Pons (L) | 1.78 |
|  | 42 | 86 | Retrosplenial area lateral agranular part (R) | 2.17 |
|  | 41 | 83 | Olfactory areas (R) | 2.04 |
|  | 40 | 64 | Olfactory areas (L) | 2.26 |
|  | 39 | 38 | Supplemental somatosensory area (L) | 2.06 |
|  |  |  | Visceral area (L) | 1.92 |
|  | 38 | 38 | Main olfactory bulb (R) | 2.09 |
|  | 37 | 36 | Retrosplenial area dorsal part (R) | 1.92 |
|  | 36 | 23 | Piriform area (L) | 1.94 |
|  |  |  | Agranular insular area posterior part (L) | 1.78 |
|  | 35 | 19 | Olfactory nerve layer of olfactory bulb (L) | 1.95 |
|  | 34 | 14 | Cerebellum crus II (R) | 2.60 |
|  | 33 | 13 | Cerebellum lobules IV-V (R) | 1.80 |
|  | 32 | 9 | Primary visual area (L) | 2.23 |
|  | 31 | 7 | Main olfactory bulb (L) | 1.82 |
|  | 30 | 5 | Hippocampus CA3 (L) | 1.76 |
|  | 27 | 3 | Alveus (R) | 1.73 |
|  | 25 | 3 | Anteromedial visual area (L) | 1.76 |
|  | 24 | 2 | Posteromedial visual area (L) | 1.69 |
|  | 23 | 2 | Agranular insular area dorsal part (R) | 1.76 |
|  | 22 | 2 | Dorsomedial hypothalamic nucleus (R) | 1.69 |
|  | 20 | 2 | Laterointermediate visual area (R) | 1.70 |
|  | 19 | 2 | Primary somatosensory area – barrel field (R) | 1.70 |
|  | 18 | 1 | Medulla (L) | 1.72 |
|  | 17 | 1 | Primary somatosensory area trunk layer (L) | 1.69 |
|  | 14 | 1 | Cerebellum paraflocculus (R) | 1.73 |
|  | 12 | 1 | Primary somatosensory area barrel field (L) | 1.79 |
|  | 10 | 1 | Secondary motor area (R) | 1.67 |
|  | 9 | 1 | Cerebellum crus I (R) | 1.67 |
|  | 8 | 1 | Laterointermediate visual area (L) | 1.69 |
|  | 6 | 1 | Entorhinal area medial part dorsal (R) | 1.68 |

**Table S3 Legend:** Cluster number, cluster size in voxels, and brain regions with peak t-values for clusters with significantly (TFCE and FWE-corrected p < 0.05) lower radial diffusivity (RD) in C58 mice compared to C57 mice based on a post hoc t-test conducted in FSL randomise. Cluster information extracted using the FSL Cluster tool. Clusters with maxima at duplicate regions not shown. FWE = familywise error rate. TFCE = threshold-free cluster enhancement.

**Table S4**. Clusters showing significantly lower MD in adult C58 mice.

| Diffusion Metric | Cluster  No. | Cluster Voxels | Location (Hemisphere) | T-statistic |
| --- | --- | --- | --- | --- |
| MD | 28 | 2546754 | Median preoptic nucleus (L) | 13.40 |
|  |  |  | Arcuate hypothalamic nucleus (L) | 13.20 |
|  |  |  | Arcuate hypothalamic nucleus (R) | 12.20 |
|  |  |  | Periventricular hypothalamic nucleus intermediate part (R) | 11.20 |
|  |  |  | Cerebellum flocculus (L) | 11.20 |
|  |  |  | Arbor vitae | 11.10 |
|  | 27 | 124 | Olfactory areas (R) | 2.14 |
|  | 26 | 122 | Paraflocculus (R) | 1.99 |
|  | 24 | 51 | Pons (L) | 1.89 |
|  | 23 | 17 | Cerebellum crus II (R) | 2.56 |
|  | 22 | 7 | Anterior visual area (L) | 1.80 |
|  | 21 | 7 | Agranular insular area posterior part (L) | 1.80 |
|  | 20 | 6 | Olfactory areas (L) | 1.88 |
|  | 19 | 5 | Primary somatosensory area mouth layer (R) | 1.77 |
|  | 18 | 5 | Primary motor area (L) | 1.71 |
|  | 17 | 4 | Inferior colliculus external nucleus (L) | 1.80 |
|  | 16 | 3 | Primary visual area (L) | 1.90 |
|  | 14 | 3 | Piriform area (L) | 1.78 |
|  | 13 | 2 | Gustatory areas (L) | 1.67 |
|  | 12 | 2 | Supplemental somatosensory area layer (R) | 1.68 |
|  | 10 | 1 | Primary somatosensory area barrel field (L) | 1.80 |
|  | 7 | 1 | Cerebellum crus I (L) | 1.70 |
|  | 6 | 1 | Main olfactory bulb (R) | 1.77 |
|  | 4 | 1 | Olfactory nerve layer of olfactory bulb (L) | 1.68 |
|  | 3 | 1 | Main olfactory bulb (L) | 1.68 |

**Table S4 Legend:** Cluster number, cluster size in voxels, and brain regions with peak t-values for clusters with significantly (TFCE and FWE-corrected p < 0.05) lower mean diffusivity (MD) in C58 mice compared to C57 mice based on a post hoc t-test conducted in FSL randomise. Cluster information extracted using the FSL Cluster tool. Clusters with maxima at duplicate regions not shown. FWE = familywise error rate. TFCE = threshold-free cluster enhancement.

**Table S5.** Clusters showing significantly lower FA in adult female mice exposed to environmental enrichment.

| Diffusion Metric | Cluster  No. | Cluster Voxels | Location (Hemisphere) | T-statistic |
| --- | --- | --- | --- | --- |
| FA | 1 | 53995 | Agranular insular area posterior part (L) | 6.78 |
|  |  |  | Primary motor area (L) | 5.88 |
|  |  |  | Primary somatosensory area nose layer (L) | 5.45 |
|  |  |  | Agranular insular area ventral part (L) | 5.08 |

**Table S5 Legend:** Cluster number, cluster size in voxels, and brain regions with peak t-values for clusters with significantly (TFCE and FWE-corrected p < 0.05) lower fractional anisotropy (FA) in EE-housed females compared to SH-housed females based on a post hoc t-test conducted in FSL randomise. Cluster information extracted using the FSL Cluster tool. EE = environmental enrichment. FWE = familywise error rate. SH = standard-housed. TFCE = threshold-free cluster enhancement.

**Table S6.** Clusters showing significantly lower FA in adult EE-housed C57 females compared to SH-housed C57 females.

| Diffusion Metric | Cluster  No. | Cluster Voxels | Location (Hemisphere) | T-statistic |
| --- | --- | --- | --- | --- |
| FA | 1 | 443042 | Bed nucleus of the stria terminalis (R) | 8.80 |
|  |  |  | Dorsolateral striatum (R) | 8.78 |
|  |  |  | Agranular insular area posterior part (L) | 8.69 |
|  |  |  | Olfactory tubercle (R) | 7.95 |
|  |  |  | Main olfactory bulb (R) | 7.46 |
|  |  |  | Thalamus – unspecified (L) | 7.22 |

**Table S6 Legend:** Cluster number, cluster size in voxels, and brain regions with peak t-values for clusters with significantly (TFCE and FWE-corrected p < 0.05) lower fractional anisotropy (FA) in adult EE-housed C57 female mice compared to SH-housed C57 female mice based on a post hoc t-test conducted in FSL randomise. Cluster information extracted using the FSL Cluster tool. Clusters with maxima at duplicate regions not shown. EE = environmental enrichment. FWE = familywise error rate. SH = standard-housed. TFCE = threshold-free cluster enhancement.

**Table S7.** Clusters showing significantly lower FA in adult EE-housed C58 males compared to SH-housed C57 males.

| Diffusion Metric | Cluster  No. | Cluster Voxels | Location (Hemisphere) | T-statistic |
| --- | --- | --- | --- | --- |
| FA | 3 | 92758 | Cerebellum Interposed nucleus (R) | 11.60 |
|  |  |  | Parvicellular reticular nucleus (R) | 11.50 |
|  |  |  | Paraflocculus (R) | 11.20 |
|  |  |  | Spinal nucleus of the trigeminal (R) | 10.40 |
|  | 2 | 16491 | Medial amygdala nucleus (R) | 8.49 |
|  |  |  | Hippocampus CA1 (R) | 7.67 |
|  |  |  | Optic radiation (R) | 7.59 |
|  |  |  | Corpus callosum splenium (R) | 6.96 |
|  | 1 | 6979 | Third ventricle | 11.30 |
|  |  |  | Periaqueductal gray (L) | 11.20 |
|  |  |  | Midbrain reticular nucleus (L) | 8.18 |
|  |  |  | Dorsal hippocampal commissure (R) | 7.13 |

**Table S7 Legend:** Cluster number, cluster size in voxels, and brain regions with peak t-values for clusters with significantly (TFCE and FWE-corrected p < 0.05) lower fractional anisotropy (FA) in adult EE-housed C58 male mice compared to SH-housed C57 male mice based on a post hoc t-test conducted in FSL randomise. Cluster information extracted using the FSL Cluster tool. Clusters with maxima at duplicate regions not shown. EE = environmental enrichment. FWE = familywise error rate. SH = standard-housed. TFCE = threshold-free cluster enhancement.

**Table S8.** Clusters showing significantly lower FA in adult EE-housed C58 males compared to EE-housed C57 males.

| Diffusion Metric | Cluster  No. | Cluster Voxels | Location (Hemisphere) | T-statistic |
| --- | --- | --- | --- | --- |
| FA | 1 | 971050 | Dorsomedial striatum (L) | 10.90 |
|  |  |  | Paraflocculus (L) | 10.60 |
|  |  |  | Arbor vitae | 10.50 |
|  |  |  | Lateral ventricle (L) | 10.30 |

**Table S8 Legend:** Cluster number, cluster size in voxels, and brain regions with peak t-values for clusters with significantly (TFCE and FWE-corrected p < 0.05) lower fractional anisotropy (FA) in adult EE-housed C58 male mice compared to EE-housed C57 male mice based on a post hoc t-test conducted in FSL randomise. Cluster information extracted using the FSL Cluster tool. EE = environmental enrichment. FWE = familywise error rate. SH = standard-housed. TFCE = threshold-free cluster enhancement.

**Table S9**. Clusters showing significantly lower FA in adult SH-housed C58 males compared to EE-housed C57 males.

| Diffusion Metric | Cluster  No. | Cluster Voxels | Location (Hemisphere) | T-statistic |
| --- | --- | --- | --- | --- |
| FA | 1 | 417476 | Central amygdala nucleus capsular part (L) | 13.20 |
|  |  |  | Nucleus accumbens (L) | 11.00 |
|  |  |  | Bed nucleus of the stria terminalis (R) | 10.30 |
|  |  |  | Pontine gray (R) | 9.61 |

**Table S9 Legend:** Cluster number, cluster size in voxels, and brain regions with peak t-values for clusters with significantly (TFCE and FWE-corrected p < 0.05) lower fractional anisotropy (FA) in adult SH-housed C58 male mice compared to EE-housed C57 male mice based on a post hoc t-test conducted in FSL randomise. Cluster information extracted using the FSL Cluster tool. EE = environmental enrichment. FWE = familywise error rate. SH = standard-housed. TFCE = threshold-free cluster enhancement.

**Table S10.** Clusters showing significantly lower FA in adult EE-housed C58 females compared to SH-housed C57 females.

| Diffusion Metric | Cluster  No. | Cluster Voxels | Location (Hemisphere) | T-statistic |
| --- | --- | --- | --- | --- |
| FA | 131 | 806386 | Posterior commissure (L) | 13.60 |
|  |  |  | Gustatory areas (L) | 13.30 |
|  |  |  | Bed nucleus of the stria terminalis (R) | 11.60 |
|  |  |  | Nucleus accumbens (R) | 11.20 |
|  |  |  | Posterior commissure (R) | 10.70 |
|  | 130 | 3080 | Periaqueductal gray (L) | 4.05 |
|  |  |  | Periaqueductal gray (R) | 3.87 |
|  |  |  | Midbrain (R) | 3.57 |
|  |  |  | Midbrain (L) | 3.50 |
|  | 129 | 2134 | Hippocampus CA1 (L) | 4.33 |
|  |  |  | Dentate gyrus molecular layer (L) | 3.36 |
|  |  |  | Dentate gyrus granule cell layer (L) | 3.16 |
|  |  |  | Hippocampus CA3 (L) | 3.02 |
|  |  |  | Lateral posterior thalamic nucleus (L) | 2.05 |
|  | 128 | 1744 | Entorhinal area medial part dorsal (R) | 3.16 |
|  | 127 | 1466 | Primary motor area (R) | 2.96 |
|  |  |  | Primary somatosensory area trunk layer (R) | 2.93 |
|  |  |  | Primary somatosensory area lower limb (R) | 2.82 |
|  |  |  | Primary somatosensory area upper limb (R) | 2.60 |
|  |  |  | Retrosplenial area lateral agranular area (R) | 2.43 |
|  | 126 | 1156 | Nucleus of the lateral olfactory tract (R) | 3.14 |
|  |  |  | Cortical amygdala area anterior part (R) | 3.01 |
|  |  |  | Basomedial amygdala nucleus anterior part (R) | 2.95 |
|  |  |  | Medial amygdala nucleus (R) | 2.73 |
|  | 125 | 808 | Parasubiculum (R) | 3.47 |
|  | 124 | 760 | Primary visual area (L) | 4.10 |
|  |  |  | Retrosplenial area lateral agranular part (L) | 3.06 |
|  | 123 | 691 | Cerebellum declive | 4.32 |
|  | 122 | 639 | Superior colliculus motor related (L) | 3.58 |
|  |  |  | Brachium of the superior colliculus (L) | 2.94 |
|  | 121 | 628 | Cortical amygdala area posterior part (R) | 3.43 |
|  |  |  | Posterior amygdala nucleus (R) | 2.65 |
|  | 120 | 493 | Dentate gyrus molecular layer (R) | 3.38 |
|  | 119 | 401 | Paraflocculus (R) | 4.54 |
|  | 118 | 373 | Superior colliculus optic layer (R) | 4.33 |
|  | 117 | 242 | Olfactory areas (L) | 2.58 |
|  | 116 | 235 | Secondary motor area (R) | 2.17 |
|  |  |  | Anterior cingulate area dorsal part (R) | 2.04 |
|  | 115 | 205 | Midbrain (R) | 4.11 |
|  |  |  | Inferior colliculus external nucleus (R) | 3.38 |
|  | 114 | 180 | Cerebellum lobules IV-V | 3.15 |
|  | 113 | 174 | Lateral habenula (L) | 3.02 |
|  | 111 | 143 | Primary somatosensory area upper limb (L) | 2.84 |
|  |  |  | Primary somatosensory area lower limb (L) | 2.22 |
|  | 110 | 133 | Main olfactory bulb (R) | 3.72 |
|  | 109 | 128 | Lateral lemniscus (L) | 2.52 |
|  | 107 | 111 | Parvicellular reticular nucleus (L) | 2.09 |
|  | 106 | 110 | Nucleus accumbens (L) | 2.45 |
|  | 104 | 85 | Lateral posterior thalamic nucleus (R) | 1.84 |
|  | 101 | 71 | Secondary motor area (L) | 2.01 |
|  | 100 | 65 | Internal capsule (L) | 2.60 |
|  | 99 | 57 | Cerebellum crus I (R) | 2.40 |
|  | 98 | 48 | Striatum (L) | 2.06 |
|  | 96 | 45 | External capsule (R) | 2.16 |
|  | 95 | 44 | Piriform area (L) | 2.03 |
|  | 92 | 37 | Cerebellum crus I (L) | 2.83 |
|  | 91 | 37 | Arbor vitae | 1.99 |
|  | 90 | 36 | Ventral posterolateral thalamic nucleus (R) | 2.02 |
|  | 89 | 35 | Postpiriform transition area (R) | 3.28 |
|  | 86 | 25 | Orbital area medial part (L) | 1.93 |
|  | 85 | 25 | Superior cerebellar peduncle (L) | 1.90 |
|  | 84 | 23 | Piriform area (R) | 2.30 |
|  | 83 | 22 | Lateral dorsal thalamic nucleus (L) | 2.10 |
|  | 82 | 22 | Cerebellum copula pyramidis (R) | 1.88 |
|  | 81 | 21 | Ethmoid thalamic nucleus (R) | 1.93 |
|  | 80 | 20 | Cerebellum paramedian lobule (R) | 1.88 |
|  | 78 | 19 | Midbrain reticular nucleus (L) | 1.95 |
|  | 76 | 17 | Primary visual area (R) | 2.25 |
|  | 75 | 17 | Midbrain (L) | 1.96 |
|  | 74 | 16 | Dorsal hippocampal commissure (R) | 1.86 |
|  | 71 | 15 | Posterior auditory area (L) | 1.85 |
|  | 68 | 11 | Lateral olfactory tract (R) | 2.33 |
|  | 67 | 11 | Entorhinal area lateral part (R) | 2.18 |
|  | 65 | 11 | Supplemental somatosensory area (R) | 2.17 |
|  | 64 | 11 | Substantia innominata (L) | 2.03 |
|  | 63 | 10 | Columns of the fornix | 2.14 |
|  | 62 | 9 | Anterior olfactory nucleus (L) | 1.71 |
|  | 60 | 8 | Cerebellum simple lobule (L) | 1.84 |
|  | 59 | 8 | Anteromedial thalamic nucleus dorsal part (L) | 1.97 |
|  | 58 | 8 | Cerebellum flocculus (L) | 1.88 |
|  | 57 | 7 | Anterior olfactory nucleus (R) | 1.77 |
|  | 52 | 7 | Cerebellum folium-tuber vermis | 1.70 |
|  | 50 | 6 | Cerebellum uvula | 1.95 |
|  | 49 | 6 | Lateral septal nucleus caudodorsal (L) | 1.71 |
|  | 47 | 6 | Cerebellum crus II (R) | 1.78 |
|  | 45 | 5 | Cerebellum simple lobule (R) | 1.76 |
|  | 44 | 5 | Stria medullaris (L) | 2.13 |
|  | 42 | 5 | Ventral anterolateral thalamic complex | 1.84 |
|  | 41 | 5 | Anterior cingulate area dorsal part (L) | 1.77 |
|  | 40 | 5 | Substantia innominata (R) | 1.90 |
|  | 39 | 4 | Hippocampal CA3 (R) | 1.79 |
|  | 38 | 4 | Hippocampal CA1 (R) | 1.78 |
|  | 37 | 4 | Olfactory nerve layer of the olfactory bulb (R) | 1.85 |
|  | 36 | 4 | Primary somatosensory area nose layer (L) | 1.74 |
|  | 35 | 4 | Reticular thalamic nucleus (L) | 1.74 |
|  | 33 | 3 | Olfactory tubercle (L) | 1.79 |
|  | 32 | 3 | Posterior amygdala nucleus (R) | 1.76 |
|  | 30 | 3 | Piriform area (L) | 2.12 |
|  | 28 | 3 | Dorsal auditory area (R) | 1.68 |
|  | 27 | 3 | Perirhinal area (R) | 1.87 |
|  | 23 | 2 | Primary somatosensory area barrel field (L) | 1.70 |
|  | 18 | 1 | Olfactory nerve layer of the olfactory bulb (L) | 1.68 |
|  | 15 | 1 | Lateral olfactory tract (R) | 1.81 |
|  | 6 | 1 | Supplemental somatosensory area (L) | 1.69 |
|  | 1 | 1 | Peripeduncular nucleus (L) | 1.68 |

**Table S10 Legend:** Cluster number, cluster size in voxels, and brain regions with peak t-values for clusters with significantly (TFCE and FWE-corrected p < 0.05) lower fractional anisotropy (FA) in adult EE-housed C58 female mice compared to SH-housed C57 female mice based on a post hoc t-test conducted in FSL randomise. Cluster information extracted using the FSL Cluster tool. Clusters with maxima at duplicate regions not shown. EE = environmental enrichment. FWE = familywise error rate. SH = standard-housed. TFCE = threshold-free cluster enhancement.

**Table S11.** Clusters showing significantly lower FA in adult SH-housed C58 females compared to EE-housed C57 females.

| Diffusion Metric | Cluster  No. | Cluster Voxels | Location (Hemisphere) | T-statistic |
| --- | --- | --- | --- | --- |
| FA | 1 | 63117 | Dorsomedial striatum (R) | 11.90 |
|  |  |  | Dorsomedial striatum (L) | 10.30 |
|  |  |  | Lateral ventricle (L) | 10.30 |

**Table S11 Legend:** Cluster number, cluster size in voxels, and brain regions with peak t-values for clusters with significantly (TFCE and FWE-corrected p < 0.05) lower fractional anisotropy (FA) in adult SH-housed C58 female mice compared to EE-housed C57 female mice based on a post hoc t-test conducted in FSL randomise. Cluster information extracted using the FSL Cluster tool. EE = environmental enrichment. FWE = familywise error rate. SH = standard-housed. TFCE = threshold-free cluster enhancement.

**Table S12.** Clusters showing significantly lower FA in adult SH-housed C58 females compared to SH-housed C57 females.

| Diffusion Metric | Cluster  No. | Cluster Voxels | Location (Hemisphere) | T-statistic |
| --- | --- | --- | --- | --- |
| FA | 90 | 711478 | Lateral septal nucleus ventral part (R) | 10.50 |
|  |  |  | Pontine reticular nucleus (R) | 9.32 |
|  |  |  | Dorsomedial striatum (L) | 8.78 |
|  |  |  | Parafascicular thalamic nucleus (L) | 8.73 |
|  |  |  | Periaqueductal gray (L) | 8.63 |
|  |  |  | Lateral ventricle (R) | 8.57 |
|  | 89 | 2996 | Primary somatosensory area -unassigned (R) | 3.10 |
|  |  |  | Primary somatosensory area – nose layer (R) | 2.94 |
|  |  |  | Primary somatosensory area – upper limb (R) | 2.79 |
|  | 88 | 20211 | Cerebellum copula pyramidis (R) | 2.99 |
|  |  |  | Cerebellum uvula (R) | 2.88 |
|  |  |  | Cerebellum pyramus (R) | 2.84 |
|  | 87 | 1719 | Medial vestibular nucleus (L) | 5.34 |
|  |  |  | Parvicellular reticular nucleus (L) | 2.81 |
|  | 86 | 834 | Anterior cingulate area dorsal part (R) | 3.87 |
|  | 85 | 573 | Hippocampus CA1 (R) | 2.16 |
|  |  |  | Subiculum (R) | 2.01 |
|  |  |  | Hippocampus CA3 (R) | 2.00 |
|  |  |  | Dentate gyrus granule cell layer (R) | 1.98 |
|  |  |  | Dentate gyrus molecular layer (R) | 1.97 |
|  | 84 | 473 | Globus pallidus external segment (L) | 2.91 |
|  |  |  | Ventrolateral striatum (L) | 2.37 |
|  | 83 | 462 | Lateral visual area (L) | 2.87 |
|  |  |  | Laterointermediate visual area (L) | 2.11 |
|  |  |  | Posterior auditory area (L) | 2.01 |
|  |  |  | Anterolateral visual area (L) | 1.85 |
|  | 82 | 356 | Olfactory areas (L) | 2.92 |
|  |  |  | Agranular insular area ventral part (L) | 2.62 |
|  |  |  | Endopiriform nucleus dorsal part (L) | 2.29 |
|  |  |  | Piriform area (L) | 2.01 |
|  | 81 | 332 | Cerebellum folium-tuber vermis (L) | 2.94 |
|  |  |  | Arbor vitae | 2.71 |
|  | 80 | 317 | Hippocampus CA1 (L) | 2.15 |
|  |  |  | Corpus callosum splenium | 1.93 |
|  | 79 | 274 | Internal capsule (L) | 3.65 |
|  | 78 | 242 | Olfactory nerve layer of olfactory bulb (R) | 2.50 |
|  |  |  | Main olfactory bulb (R) | 1.96 |
|  | 77 | 192 | Ventral posterolateral thalamic nucleus (L) | 3.57 |
|  | 76 | 170 | Lateral olfactory tract L) | 2.40 |
|  | 75 | 167 | Piriform area (R) | 2.87 |
|  | 74 | 130 | Primary somatosensory area barrel field (L) | 2.87 |
|  | 73 | 117 | Hippocampus CA3 (L) | 2.02 |
|  | 71 | 102 | Cortical amygdala area posterior part (R) | 2.15 |
|  | 70 | 91 | Dorsolateral striatum (R) | 2.01 |
|  | 69 | 82 | Entorhinal area medial part dorsal (R) | 2.69 |
|  |  |  | Postpiriform transition area (R) | 1.74 |
|  | 68 | 79 | Pontine reticular nucleus (L) | 2.37 |
|  |  |  | Paranigral nucleus (L) | 2.35 |
|  |  |  | Medial lemniscus (L) | 2.10 |
|  | 66 | 59 | Substantia innominata (L) | 2.18 |
|  | 65 | 59 | Primary motor area (R) | 1.93 |
|  | 64 | 57 | Dentate gyrus molecular layer (L) | 2.07 |
|  | 63 | 50 | Ventral striatum (L) | 2.05 |
|  | 60 | 41 | Secondary motor area (R) | 2.09 |
|  | 59 | 40 | Supplemental somatosensory area (L) | 2.13 |
|  | 57 | 23 | Parabrachial nucleus (R) | 1.88 |
|  | 56 | 23 | Anterior amygdala area (R) | 2.47 |
|  | 55 | 21 | Intermediate reticular nucleus (R) | 2.00 |
|  | 51 | 12 | Superior colliculus superficial gray layer (L) | 1.90 |
|  | 50 | 11 | Anterior olfactory nucleus (R) | 1.92 |
|  | 49 | 10 | Entorhinal area lateral part (R) | 1.98 |
|  | 48 | 10 | Cerebellum simple lobule (L) | 1.96 |
|  | 47 | 10 | Orbital area lateral part (L) | 1.86 |
|  | 45 | 9 | Frontal pole (L) | 1.74 |
|  | 44 | 9 | Superior colliculus motor-related intermediate layer (L) | 1.78 |
|  | 42 | 8 | Cerebellum paraflocculus (R) | 1.95 |
|  | 40 | 8 | Agranular insular area dorsal part (R) | 1.76 |
|  | 39 | 8 | Prelimbic area (R) | 1.83 |
|  | 38 | 7 | Anteromedial thalamic nucleus dorsal part (R) | 1.71 |
|  | 37 | 7 | Olfactory nerve layer of olfactory bulb (L) | 2.06 |
|  | 36 | 7 | External cuneate nucleus (R) | 1.90 |
|  | 34 | 6 | Internal capsule (R) | 1.83 |
|  | 33 | 5 | Basolateral amygdala nucleus posterior part (R) | 1.84 |
|  | 32 | 4 | Olfactory areas (R) | 2.06 |
|  | 30 | 4 | Lateral septal nucleus rostroventral part (R) | 1.72 |
|  | 29 | 4 | Cerebellum lobules IV-V | 1.87 |
|  | 28 | 3 | Piriform area (L) | 1.77 |
|  | 26 | 3 | Cerebellum paraflocculus (L) | 1.86 |
|  | 25 | 2 | Cerebellum crus I | 1.83 |
|  | 24 | 2 | Main olfactory bulb (L) | 1.69 |
|  | 23 | 2 | Primary somatosensory area mouth layer (R) | 1.69 |
|  | 22 | 2 | Supplemental somatosensory area (R) | 1.88 |
|  | 20 | 2 | Olfactory tubercle (L) | 1.69 |
|  | 17 | 2 | Gustatory areas (R) | 1.72 |
|  | 14 | 2 | Pons (R) | 1.76 |
|  | 10 | 1 | Nucleus of Roller (L) | 1.71 |
|  | 6 | 1 | Primary visual area (L) | 1.79 |
|  | 4 | 1 | Auditory radiation (R) | 1.71 |
|  | 2 | 1 | Orbital area lateral part (R) | 1.68 |
|  | 1 | 1 | Primary motor area (L) | 1.68 |

**Table S12 Legend:** Cluster number, cluster size in voxels, and brain regions with peak t-values for clusters with significantly (TFCE and FWE-corrected p < 0.05) lower fractional anisotropy (FA) in adult SH-housed C58 female mice compared to SH-housed C57 female mice based on a post hoc t-test conducted in FSL randomise. Cluster information extracted using the FSL Cluster tool. Clusters with maxima at duplicate regions not shown. EE = environmental enrichment. FWE = familywise error rate. SH = standard-housed. TFCE = threshold-free cluster enhancement.

**Table S13.** Clusters with a significant negative correlation between RRB and FA in the adult cohort.

| Diffusion  Metric | Cluster  No. | Cluster Voxels | Location (Hemisphere) | T-statistic | Correlation  (all mice) | Correlation (C58 only) |
| --- | --- | --- | --- | --- | --- | --- |
| FA | 1 | 56131 | Internal capsule (L) | 6.19 | -0.52 | -0.40 |
|  |  |  | External capsule (R) | 5.56 | -0.54 | -0.51 |
|  |  |  | Internal capsule (R) | 5.41 | -0.52 | -0.46 |
|  |  |  | Caudal striatum (R) | 5.15 | -0.59 | -0.51 |

**Table S13 Legend:** Cluster number, cluster size in voxels, and brain regions with peak t-values for clusters with a significant (TFCE and FWE-corrected p < 0.05) negative correlation between restricted, repetitive behavior (RRB) and fractional anisotropy (FA) based on a voxelwise correlation conducted in FSL randomise. Cluster information extracted using the FSL Cluster tool. Separate post hoc Spearman rank correlation coefficients are provided for both mouse strains combined (all mice) and C58 mice only. FWE = familywise error rate. TFCE = threshold-free cluster enhancement.

**Table S14.** Clusters with a significant positive correlation between RRB and RD in the adult cohort.

| Diffusion  Metric | Cluster  No. | Cluster Voxels | Location (Hemisphere) | T-statistic | Correlation  (all mice) | Correlation  (C58 only) |
| --- | --- | --- | --- | --- | --- | --- |
| RD | 1 | 125392 | Dorsomedial striatum (L) | 6.76 | 0.45 | 0.34 |
|  |  |  | Corpus callosum body | 6.27 | 0.49 | 0.43 |
|  |  |  | Dorsomedial striatum (R) | 6.23 | 0.56 | 0.47 |
|  |  |  | Corpus callosum genu | 6.18 | 0.33 | 0.36 |

**Table S14 Legend:** Cluster number, cluster size in voxels, and brain regions with peak t-values for clusters with a significant (TFCE and FWE-corrected p < 0.05) positive correlation between restricted, repetitive behavior (RRB) and radial diffusivity (RD) based on a voxelwise correlation conducted in FSL randomise. Cluster information extracted using the FSL Cluster tool. Separate post hoc Spearman rank correlation coefficients are provided for both mouse strains combined (all mice) and C58 mice only. FWE = familywise error rate. TFCE = threshold-free cluster enhancement.

**Table S15.** Clusters with significantly greater FA in juvenile C58 mice exposed to environmental enrichment.

| Diffusion Metric | Cluster  No. | Cluster Voxels | Location (Hemisphere) | T-statistic |
| --- | --- | --- | --- | --- |
| FA | 112 | 699942 | Entorhinal area medial part dorsal zone (L) | 9.67 |
|  |  |  | Inferior colliculus external nucleus (R) | 7.74 |
|  |  |  | Entorhinal area lateral part (R) | 7.37 |
|  |  |  | Midbrain (L) | 7.35 |
|  |  |  | Olfactory areas (R) | 7.07 |
|  | 111 | 1961 | Dentate gyrus granule cell layer (L) | 3.27 |
|  |  |  | Hippocampus CA3 (L) | 2.98 |
|  |  |  | Hippocampus CA1 (L) | 2.89 |
|  |  |  | Dentate gyrus molecular layer (L) | 2.89 |
|  | 110 | 787 | Arbor vitae | 2.66 |
|  |  |  | Cerebellum fastigial nucleus (R) | 2.52 |
|  |  |  | Cerebellum nodulus | 2.25 |
|  | 109 | 656 | Postlateral visual area (R) | 2.83 |
|  | 108 | 613 | Superior olivary complex lateral part (L) | 2.97 |
|  |  |  | Lateral lemniscus (L) | 2.19 |
|  | 107 | 588 | Agranular insular area dorsal part (R) | 2.97 |
|  |  |  | Agranular insular area ventral part (R) | 2.14 |
|  | 106 | 581 | Optic radiation (L) | 3.22 |
|  |  |  | Lateral geniculate complex thalamus (L) | 1.86 |
|  | 105 | 548 | Ventral cochlear nucleus (R) | 3.08 |
|  |  |  | Medulla (R) | 1.94 |
|  | 104 | 533 | External cuneate nucleus (R) | 2.72 |
|  |  |  | Cerebellum uvula | 2.55 |
|  |  |  | Spinal trigeminal nucleus (R) | 2.26 |
|  | 103 | 320 | Visceral area (R) | 2.79 |
|  | 102 | 288 | Cerebellum crus I (L) | 2.65 |
|  |  |  | Cerebellum simple lobule (L) | 2.61 |
|  | 101 | 281 | Retrosplenial area dorsal part (L) | 3.69 |
|  | 100 | 257 | Cerebellum simple lobule (R) | 2.28 |
|  | 99 | 203 | Superior colliculus motor related (R) | 2.53 |
|  | 98 | 139 | Secondary motor area (L) | 2.07 |
|  | 97 | 90 | Midbrain (R) | 1.98 |
|  | 96 | 86 | Retrosplenial area dorsal part (R) | 2.14 |
|  | 95 | 78 | Infracerebellar nucleus (R) | 2.48 |
|  | 94 | 67 | Retrosplenial area lateral agranular part (R) | 1.91 |
|  | 92 | 63 | Inferior cerebellar peduncle (R) | 1.94 |
|  | 91 | 61 | Primary somatosensory area mouth layer (R) | 2.00 |
|  | 90 | 57 | Substantia innominata (L) | 1.93 |
|  |  |  | Pallidum (L) | 1.76 |
|  | 88 | 46 | Dentate gyrus molecular layer (R) | 2.00 |
|  |  |  | Lateral posterior thalamic nucleus (R) | 1.95 |
|  | 86 | 38 | Tegmental reticular nucleus (L) | 2.20 |
|  | 85 | 37 | Supplemental somatosensory area (R) | 1.88 |
|  | 84 | 37 | Corpus callosum body | 1.88 |
|  | 83 | 35 | Piriform area (R) | 2.13 |
|  | 82 | 33 | Agranular area dorsal part (L) | 2.14 |
|  | 81 | 28 | Parasubiculum (R) | 1.98 |
|  | 78 | 23 | Pontine reticular nucleus (R) | 2.02 |
|  | 76 | 19 | Medial vestibular nucleus (R) | 2.03 |
|  | 74 | 15 | Cerebellum crus II (R) | 1.91 |
|  | 73 | 15 | Main olfactory bulb (L) | 1.88 |
|  | 72 | 15 | Cerebellum lobules IV-V | 2.07 |
|  | 70 | 13 | Primary somatosensory area unassigned (R) | 1.87 |
|  | 69 | 13 | Supracallosal white matter (L) | 2.09 |
|  | 68 | 12 | Primary motor area (L) | 1.79 |
|  | 67 | 11 | Postrhinal area (R) | 2.32 |
|  | 66 | 10 | Striatum (L) | 1.88 |
|  | 65 | 10 | Spinal tract of the trigeminal nerve (R) | 1.88 |
|  | 64 | 9 | Entorhinal area medial part dorsal zone (R) | 1.80 |
|  | 63 | 9 | Central lateral thalamic nucleus (L) | 1.71 |
|  | 59 | 7 | Dorsomedial striatum (R) | 1.72 |
|  | 58 | 7 | Medial septal nucleus (L) | 1.83 |
|  | 55 | 6 | Parasubiculum (L) | 1.92 |
|  | 50 | 5 | Primary somatosensory area unassigned (L) | 1.81 |
|  | 49 | 5 | Midbrain reticular nucleus (R) | 1.73 |
|  | 48 | 5 | Cerebellum flocculus (R) | 1.72 |
|  | 47 | 5 | Lateral posterior thalamic nucleus (L) | 1.75 |
|  | 46 | 4 | Endopiriform nucleus dorsal part (R) | 1.70 |
|  | 45 | 4 | Presubiculum (R) | 1.74 |
|  | 44 | 4 | Hippocampus CA3 (R) | 1.75 |
|  | 41 | 3 | Primary somatosensory area upper limb (L) | 1.86 |
|  | 40 | 3 | Cerebellum declive | 1.77 |
|  | 38 | 2 | Ventral cochlear nucleus (R) | 1.71 |
|  | 35 | 2 | Primary visual area (L) | 1.68 |
|  | 34 | 2 | Superior colliculus zonal layer (R) | 1.76 |
|  | 33 | 2 | Olfactory tubercle (R) | 1.69 |
|  | 29 | 2 | Interposed nucleus (L) | 1.72 |
|  | 28 | 2 | Cerebellum pyramus | 1.71 |
|  | 27 | 2 | Dorsomedial striatum (L) | 1.75 |
|  | 18 | 1 | Medial longitudinal fascicle (L) | 1.67 |
|  | 16 | 1 | Cerebellum copula pyramidis (R) | 1.68 |
|  | 14 | 1 | Primary visual area (R) | 1.70 |
|  | 11 | 1 | Reticular thalamic nucleus (L) | 1.67 |
|  | 8 | 1 | Ventral postlateral thalamic nucleus (L) | 1.67 |

**Table S15 Legend:** Cluster number, cluster size in voxels, and brain regions with peak t-values for clusters with significantly (TFCE and FWE-corrected p < 0.05) greater fractional anisotropy (FA) in juvenile EE-housed C58 mice compared to SH-housed C58 mice based on a post hoc t-test conducted in FSL randomise. Cluster information extracted using the FSL Cluster tool. Clusters with maxima at duplicate regions not shown. EE = environmental enrichment. FWE = familywise error rate. SH = standard-housed. TFCE = threshold-free cluster enhancement.

**Table S16.** Clusters with significantly greater AD in juvenile C58 mice exposed to environmental enrichment.

| Diffusion Metric | Cluster  No. | Cluster Voxels | Location (Hemisphere) | T-statistic |
| --- | --- | --- | --- | --- |
| FA | 39 | 776017 | Anterior hypothalamic nucleus (R) | 7.49 |
|  |  |  | Ventral tegmental area (L) | 7.23 |
|  |  |  | Ventral auditory area (R) | 6.78 |
|  |  |  | Hippocampus CA1 (R) | 6.38 |
|  |  |  | Cerebellum simple lobule (R) | 6.37 |
|  |  |  | Dorsal auditory area (R) | 6.27 |
|  | 38 | 1078 | Posterolateral visual area (L) | 3.88 |
|  |  |  | Primary visual area (L) | 3.13 |
|  |  |  | Lateral visual area (L) | 2.20 |
|  | 37 | 368 | Cerebellum paraflocculus (L) | 2.87 |
|  | 36 | 261 | Ventrolateral striatum (L) | 2.12 |
|  | 35 | 216 | Supplemental somatosensory area (L) | 2.21 |
|  | 34 | 200 | Cerebellum crus II (R) | 3.34 |
|  |  |  | Cerebellum declive | 2.73 |
|  | 32 | 158 | Entorhinal area medial part dorsal zone (R) | 2.58 |
|  | 31 | 120 | Striatum (L) | 2.25 |
|  | 30 | 26 | Entorhinal area lateral part (L) | 1.87 |
|  | 29 | 23 | Arbor vitae | 2.22 |
|  | 28 | 17 | Cerebellum nodulus | 1.87 |
|  | 26 | 16 | Primary motor area (R) | 1.92 |
|  | 24 | 12 | Piriform area (R) | 1.99 |
|  |  |  | Piriform-amygdala area (R) | 1.83 |
|  | 22 | 8 | Stria medullaris (R) | 1.74 |
|  | 21 | 8 | Cingulum bundle (R) | 2.12 |
|  | 20 | 7 | Postrhinal area (L) | 1.80 |
|  | 19 | 6 | Superior colliculus zonal layer (L) | 1.76 |
|  | 16 | 4 | Agranular insular area posterior part (R) | 1.69 |
|  | 14 | 3 | Cerebellum copula pyramidis (R) | 1.69 |
|  | 13 | 3 | Ventromedial striatum (R) | 1.72 |
|  | 12 | 3 | Primary somatosensory area barrel field (L) | 1.70 |
|  | 10 | 1 | Olfactory tubercle (R) | 1.68 |
|  | 8 | 1 | Superior colliculus motor area (R) | 1.70 |
|  | 7 | 1 | Primary somatosensory area nose layer (L) | 1.68 |
|  | 6 | 1 | Cortical amygdala posterior part (R) | 1.80 |
|  | 5 | 1 | Alveus (R) | 1.68 |
|  | 4 | 1 | Hypothalamus (L) | 1.70 |
|  | 3 | 1 | Orbital area medial part (L) | 1.68 |
|  | 2 | 1 | Supplemental somatosensory area (R) | 1.68 |
|  | 1 | 1 | Midbrain reticular nucleus (L) | 1.68 |

**Table S16 Legend:** Cluster number, cluster size in voxels, and brain regions with peak t-values for clusters with significantly (TFCE and FWE-corrected p < 0.05) greater axial diffusivity (AD) in juvenile EE-housed C58 mice compared to SH-housed C58 mice based on a post hoc t-test conducted in FSL randomise. Cluster information extracted using the FSL Cluster tool. Clusters with maxima at duplicate regions not shown. EE = environmental enrichment. FWE = familywise error rate. SH = standard-housed. TFCE = threshold-free cluster enhancement.

**Table S17.** Clusters with a significant negative correlation between RRB and FA in the juvenile cohort.

| Diffusion Metric | Cluster  No. | Cluster Voxels | Location (Hemisphere) | T-statistic | Correlation |
| --- | --- | --- | --- | --- | --- |
| FA | 51 | 263526 | Entorhinal area medial part dorsal zone (L) | 8.37 | -0.77 |
|  |  |  | Principal sensory nucleus of the trigeminal nerve (R) | 7.91 | -0.45 |
|  |  |  | Superior colliculus motor related (R) | 6.80 | -0.58 |
|  |  |  | Inferior cerebellar peduncle (R) | 6.75 | -0.53 |
|  |  |  | Ventral cochlear nucleus (R) | 6.63 | -0.52 |
|  | 50 | 72758 | Diagonal band nucleus (R) | 13.6 | -0.73 |
|  |  |  | Optic tract (R) | 6.65 | -0.35 |
|  |  |  | Diagonal band nucleus (L) | 6.08 | -0.70 |
|  |  |  | Nucleus accumbens (R) | 5.72 | -0.81 |
|  | 49 | 3858 | Primary somatosensory area (R) | 3.21 | -0.67 |
|  |  |  | Supplemental somatosensory area (R) | 2.99 | -0.48 |
|  | 48 | 2473 | Hippocampus CA1 (L) | 5.20 | -0.54 |
|  |  |  | Alveus (L) | 3.62 | -0.78 |
|  |  |  | Corpus callosum posterior forceps | 3.50 | -0.68 |
|  | 47 | 2242 | Optic tract (L) | 4.11 | -0.26 |
|  |  |  | Lateral hypothalamic area (L) | 2.87 | -0.35 |
|  | 46 | 1417 | Reticular thalamic nucleus (R) | 3.96 | -0.48 |
|  |  |  | Ventral posterolateral thalamic nucleus (R) | 3.50 | -0.01 |
|  |  |  | External medullary lamina of the thalamus (R) | 3.16 | -0.34 |
|  | 45 | 1150 | Hippocampus CA3 (L) | 3.49 | -0.61 |
|  |  |  | Stria terminalis (L) | 2.80 | -0.33 |
|  |  |  | Central amygdala nucleus lateral part (L) | 2.48 | -0.19 |
|  | 44 | 1017 | Intermediate reticular nucleus (R) | 3.55 | -0.70 |
|  |  |  | Magnocellular reticular nucleus (R) | 2.89 | -0.40 |
|  |  |  | Parvicellular reticular nucleus (R) | 2.59 | -0.77 |
|  |  |  | Gigantocellular reticular nucleus (R) | 2.59 | -0.45 |
|  |  |  | Linear nucleus of the medulla (R) | 2.01 | -0.49 |
|  | 43 | 692 | Arbor vitae | 5.85 | -0.76 |
|  |  |  | Cerebellum paramedian lobule (L) | 2.86 | -0.67 |
|  |  |  | Cerebellum interposed nucleus (L) | 2.59 | -0.52 |
|  |  |  | Cerebellum copula pyramidis (L) | 2.28 | -0.57 |
|  | 42 | 617 | Pons (R) | 3.20 | -0.62 |
|  |  |  | Pedunculopontine nucleus (R) | 2.91 | -0.75 |
|  |  |  | Midbrain reticular nucleus (R) | 2.69 | -0.47 |
|  |  |  | Pontine reticular nucleus caudal part (R) | 1.97 | -0.63 |
|  |  |  | Pontine reticular nucleus (R) | 1.89 | -0.33 |
|  | 41 | 447 | Visceral area (R) | 3.08 | -0.40 |
|  | 40 | 381 | Cerebellum lobules IV-V | 3.22 | -0.80 |
|  | 38 | 169 | Dentate gyrus molecular layer (L) | 2.10 | -0.58 |
|  |  |  | Lateral posterior thalamic nucleus (L) | 1.90 | -0.36 |
|  | 37 | 100 | Cerebellum simple lobule (R) | 2.25 | -0.82 |
|  | 36 | 90 | Central amygdala nucleus capsular part (R) | 2.17 | -0.72 |
|  |  |  | Central amygdala nucleus medial part (R) | 2.08 | -0.56 |
|  | 35 | 86 | Superior colliculus zonal layer (L) | 2.49 | -0.52 |
|  | 34 | 66 | Mediodorsal thalamic nucleus (L) | 2.03 | -0.62 |
|  | 32 | 42 | Caudal striatum (L) | 2.12 | -0.41 |
|  | 31 | 35 | Central lateral thalamic nucleus (L) | 1.99 | -0.58 |
|  | 30 | 34 | Hippocampal-amygdala transition area (L) | 1.96 | -0.38 |
|  | 29 | 22 | Inferior cerebellar peduncle (L) | 2.08 | -0.16 |
|  | 28 | 20 | Dorsal nucleus raphe (L) | 1.88 | -0.57 |
|  | 26 | 12 | Primary visual area (L) | 1.86 | -0.72 |
|  | 25 | 12 | Corpus callosum body | 1.73 | -0.39 |
|  |  |  | Supracallosal cerebral white matter (R) | 1.72 | -0.41 |
|  | 24 | 11 | Superior colliculus optic layer (L) | 1.77 | -0.69 |
|  |  |  | Superior colliculus motor related (L) | 1.75 | -0.74 |
|  | 23 | 7 | Parabrachial nucleus (R) | 2.13 | -0.29 |
|  | 21 | 5 | Medial vestibular nucleus (L) | 1.71 | -0.74 |
|  |  |  | Nucleus prepositus (L) | 1.68 | -0.57 |
|  | 20 | 5 | Pontine central gray (L) | 1.77 | -0.66 |
|  | 18 | 4 | Corpus callosum anterior forceps | 1.75 | -0.81 |
|  | 15 | 3 | Spinal nucleus of the trigeminal (R) | 1.71 | -0.80 |
|  | 13 | 3 | Orbital area ventrolateral part (R) | 1.83 | -0.51 |
|  | 12 | 3 | Perireunensis thalamic nucleus (R) | 1.76 | -0.20 |
|  | 9 | 2 | Substantia innominata (R) | 1.71 | -0.73 |
|  | 7 | 2 | Superior cerebellar peduncle (L) | 1.70 | -0.44 |
|  | 6 | 1 | Dorsomedial striatum (R) | 1.67 | -0.43 |
|  | 5 | 1 | Medial vestibular nucleus (R) | 1.74 | -0.53 |
|  | 1 | 1 | Primary motor area (R) | 1.67 | -0.57 |

**Table S17 Legend:** Cluster number, cluster size in voxels, and brain regions with peak t-values for clusters with a significant (TFCE and FWE-corrected p < 0.05) negative correlation between restricted, repetitive behavior (RRB) and fractional anisotropy (FA) based on a voxelwise correlation conducted in FSL randomise. Cluster information extracted using the FSL Cluster tool. Correlation coefficients are from a post hoc Spearman rank correlation. FWE = familywise error rate. TFCE = threshold-free cluster enhancement.
